# π-Stacks of radical-anionic naphthalenediimides in a metal-organic framework

**DOI:** 10.1126/sciadv.ade1383

**Published:** 2022-12-23

**Authors:** Bongkyeom Kim, Juhyung Lee, Ying-Pin Chen, Xue-Qian Wu, Joongoo Kang, Hwakyeung Jeong, Sang-Eun Bae, Jian-Rong Li, Jooyoung Sung, Jinhee Park

**Affiliations:** ^1^ Department of Physics and Chemistry, Daegu Gyeongbuk Institute of Science and Technology (DGIST), 333 Techno Jungang-daero, Dalseong-gun, Daegu 42988, Republic of Korea.; ^2^ NSF’s ChemMatCARs, The University of Chicago Argonne, Chicago, IL 60439, USA.; ^3^ Faculty of Environment and Life, Beijing University of Technology, Beijing 100124, P.R. China.; ^4^ Nuclear Chemistry Research Team, Korea Atomic Energy Research Institute, Daejeon 34057, Republic of Korea.; ^5^ Beijing Key Laboratory for Green Catalysis and Separation, Department of Chemistry and Chemical Engineering, College of Environmental and Energy Engineering, Beijing University of Technology, Beijing 100124, P.R. China.

## Abstract

Radical-ionic metal-organic frameworks (MOFs) have unique optical, magnetic, and electronic properties. These radical ions, forcibly formed by external stimulus-induced redox processes, are structurally unstable and have short radical lifetimes. Here, we report two naphthalenediimide-based (NDI-based) Ca-MOFs: DGIST-6 and DGIST-7. Neutral DGIST-6, which is generated first during solvothermal synthesis, decomposes and is converted into radical-anionic DGIST-7. Cofacial (NDI)_2_^•−^ and (NDI)_2_^2−^ dimers are effectively stabilized in DGIST-7 by electron delocalization and spin-pairing as well as dimethylammonium counter cations in their pores. Single-crystal x-ray diffractometry was used to visualize redox-associated structural transformations, such as changes in centroid-to-centroid distance. Moreover, the unusual rapid reduction of oxidized DGIST-7 into the radical anion upon infrared irradiation results in effective and reproducible photothermal conversion. This study successfully illustrated the strategic use of in situ prepared cofacial ligand dimers in MOFs that facilitate the stabilization of radical ions.

## Teaser

Radical-anionic dimers incorporated into a MOF backbone enable in-depth studies on redox-dependent structural transitions.

## INTRODUCTION

Naphthalenediimides (NDIs) are π-electron–deficient polycyclic aromatic compounds that undergo one- and two-electron reductions to their radical anions (NDI^•−^) and dianions (NDI^2−^) (*1*, *2*). The distinct optical, electronic, and magnetic properties of NDIs and their anions enable their wide applications in sensors, organic electronics, n-type semiconductors, photoelectrical devices, photothermal therapy, and catalysts (*3*–*11*). Despite the delocalization of its unpaired electron in the planar π-conjugated system, NDI^•−^ is susceptible to oxidative and collisional or aggregation-induced radical state quenching (*9*, *12*, *13*); this inherent lability limits its practical use.

A representative strategy for stabilizing NDI^•−^ involves forming NDI^•−^-based π-stacks, including dimers, oligomers, and one-dimensional (1D) stacks (*14*, *15*). Cofacial π-π interactions cause π-orbital overlap, which extends electron delocalization and stabilizes NDI^•−^. Spontaneously formed radical dimers (or monomers) are self-assembled with macrocyclic hosts such as cucurbiturils or cyclobis(paraquat-*p*-phenylene) (*13*, *16*–*19*). These supramolecular self-assemblies are less structurally restricted and, therefore, energetically favorable. However, they easily disassemble when the radical characteristics are lost. In this regard, two interlocking NDIs were designed in a covalent scaffold; however, the covalently tethered NDIs in this system exhibit a constrained cofacial arrangement with a relatively large interplanar distance because they are forced to face each other (*20*, *21*). Even this covalent NDI dimer rarely demonstrated redox-related structural transformation by single-crystal x-ray diffractometry (SCXRD), owing to its crystal instability.

Metal-organic frameworks (MOFs) are prominent platforms that accommodate NDI^•−^ π-stacks in the solid state (*22*, *23*). The structural robustness of a MOF prevents collisional quenching (*24*, *25*); furthermore, its porous structure facilitates the inclusion of charge-balancing counter cations or electron donors for stimulus-induced electron transfer (*26*, *27*). Therefore, extensive research has been conducted on the development of radical-ionic MOFs (table S1). For example, electron donors such as polyoxometalates, 1,5-/2,6-dinaphthol, 4,*N*,*N*-trimethylaniline, and tetrathiofulvalene as guest molecules were incorporated into MOF pores to stabilize the radical-anionic states (*28*–*40*). As alternatives, MOFs comprising π-electron–deficient ligands as electron acceptors and π-electron–rich ones as electron donors were prepared (*35*, *36*). In addition, cofacially stacked redox-active moieties, such as thiazolo[5,4-*d*]thiazoles and viologens, which exhibit through-space intervalence charge transfer phenomena or radical-radical interactions, were used to construct stable radical-ionic MOFs (*25*, *33*, *41*–*44*). However, thermodynamically stable inorganic secondary building units (SBUs) predominantly govern the overall structures of MOFs; consequently, there is a lesser likelihood of obtaining ligand π-stacks (*45*–*48*). Moreover, sustaining a radical-ionic state under solvothermal synthesis conditions is known to be challenging (*49*, *50*). Therefore, only a few MOFs, which were electrochemically synthesized or included interactions between the NDI units and electron donors, were synthesized in radical-anionic states, and most of the NDI-based MOFs were formed in neutral states (table S2). Consequently, external stimuli such as ultraviolet-visible (UV-vis) light, reductants/oxidants, and electric fields were needed to induce electron transfer and then afford the radical-ionic MOFs (*50*). However, the generated radical-ionic ligands were not homogeneously distributed throughout the crystals, and the rigidity of the frameworks hindered the structural optimization of the radical-ionic states. These factors have impeded the crystallographic visualization of the radical states by SCXRD; in some cases, the radical formation does not involve notable structural transition. Here, we hypothesized that solvothermal synthesis at a sufficiently high temperature in the absence of an oxidant should facilitate anion/NDI-π interactions that trigger electron transfer from the anion to the NDI to generate NDI^•−^ (*51*–*53*). Subsequently, a MOF composed of NDI radical-anionic dimers (NDI)_2_^•−^ naturally formed through strong π-π interactions is generated. Recently, our group reported the radical-anionic MOF, 150-DGIST-4, formed by the reduction of an NDI-based ligand via anion-π interaction-based electron transfer at high temperatures such as 150°C. However, because the formation of 150-DGIST-4 does not rely on the radical state of the ligand, only some of the ligands were revealed in the radical-anionic states. Many of the MOFs were synthesized at relatively low temperatures, such as 80° to 120°C, leading to the formation of neutral MOFs (tables S1 and S2). The highly ordered crystallinity of the radical-anionic MOF enables NDI- and NDI^•−^-state–dependent structural transformations to be observed by SCXRD, leading to an in-depth understanding of associated structure-property relationships (*54*). Analyzing structural transitions by SCXRD is challenging, owing to radical lability and a lack of established methods for preparing crystalline radical samples (*45*, *55*).

## RESULTS

### Radical-anionic MOF design and synthesis

**Fig. 1.**
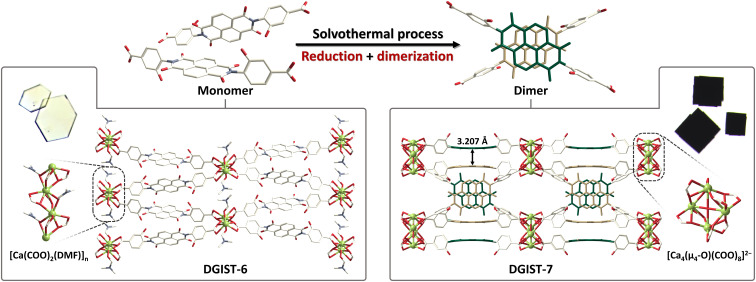
Sequential one-pot synthesis of DGIST-6 and DGIST-7. Crystal structures of DGIST-6 composed of monomeric ligands, and DGIST-7 formed by the cofacial dimerization of H_2_**L** (**L**^2−^ = *N,N′*-bis(3-hydroxybenzoate)-1,4,5,8-naphthalenediimide) during solvothermal synthesis. Atoms are depicted in the following colors: Ca, chartreuse; O, red; N, slate gray; C, ivory.

Here, H_2_**L** (**L**^2−^ = *N,N′-*bis(3-hydroxybenzoate)-1,4,5,8-naphthalenediimide) was designed to promote the cofacial self-assembly of NDI moieties into a MOF (fig. S1). Ca^2+^ was selected as the metal ion because its fixed oxidation state cannot perturb the formation of the radical-anionic ligand, (**L**^2−^)^•−^. H_2_**L** was solvothermally reacted with Ca(CH_3_COO)_2_∙H_2_O in *N*,*N-*dimethylformamide (DMF) at 150°C to initially afford colorless hexagonal crystals of DGIST-6, [Ca(**L**)(DMF)] (Fig. 1 and fig. S2). Monomeric **L**^2−^ with a neutral NDI moiety is coordinated to two different 1D [Ca(COO)_2_(DMF)]_n_ chain clusters in DGIST-6, where neighboring Ca^2+^ ions are bridged by two ligand carboxylates (μ_2_-η^2^:η^1^ mode) and an oxygen atom (μ_2_ mode) of DMF. These colorless crystals gradually disappeared with prolonged reaction time, and black cuboid crystals of DGIST-7, [Ca_4_(μ_4_-O)(**L**_2_^•−^)_2(1-n)_(**L**_2_^2−^)_2n_(DMA^+^)_4-2n_(DMA^+^)_4n_] (DMA^+^, dimethylammonium; 0 < n < 1), became dominant (figs. S3 and S4) (*56*, *57*). A ligand dimer is constructed through cofacial π-π interactions and is connected to four different [Ca_4_(μ_4_-O)(COO)_8_]^2−^ clusters in DGIST-7 (Fig. 1). The centroid-to-centroid distance between NDI planes is only 3.207 Å, which is shorter than typical π-stacking distances (*20*, *58*–*60*). This short distance is ascribable to strong π-π interactions associated with dimeric (**L**^2−^)_2_^•−^ and (**L**^2−^)_2_^2−^ that are created by the reduction of the NDI moiety. DGIST-7 is formed more slowly than DGIST-6 because formate, the electron donor, must be first generated by the decomposition of DMF. The nuclear magnetic resonance (NMR) spectrum obtained after the decomposition of the pristine DGIST-7 crystals reveals the presence of formic acid (the protonated form of formate, δ = 8.13). The ratio of formate to **L**^2−^ is determined to be 1:4. Unfortunately, the peak overlap between DMA^+^ and dimethyl sulfoxide (DMSO) impedes the quantitative analysis of DMA^+^. However, the presence of DMA^+^ (δ = 2.48) is undoubtedly disclosed. Thus, once the formate donates the electrons, the negative charges are presumably balanced with DMA^+^, and the formate radicals are released (*61*). Noteworthily, the structure of DGIST-7 is also interpretable as doubly interpenetrated monomeric ligand-based frameworks, in which one framework interacts with the other through strong π-π interactions involving NDI moieties (fig. S7).

### Analyzing the NDI^•−^ stability of DGIST-7

**Fig. 2.**
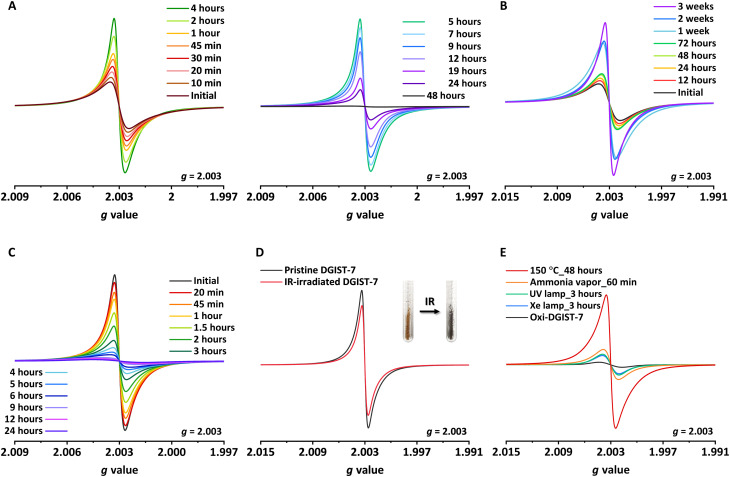
EPR spectra of DGIST-7. Monitoring NDI^•−^ in (**A**) dried pristine DGIST-7, (**B**) DMF-soaked pristine DGIST-7, and (**C**) dried DGIST-7-[Cu^2+^] as functions of air exposure time. (**D**) Comparing the EPR spectra of pristine DGIST-7 and infrared (IR)–irradiated oxi-DGIST-7. (**E**) Oxi-DGIST-7 after exposure to various stimuli. EPR spectra were recorded at a power of 1 mW, a modulation width of 0.01 mT, a time constant of 0.03 s, a conversion time of 30 s, a modulation amplitude of one time for (A) to (D) and 10 times for (E), and a temperature of 25°C.

**Fig. 3.**
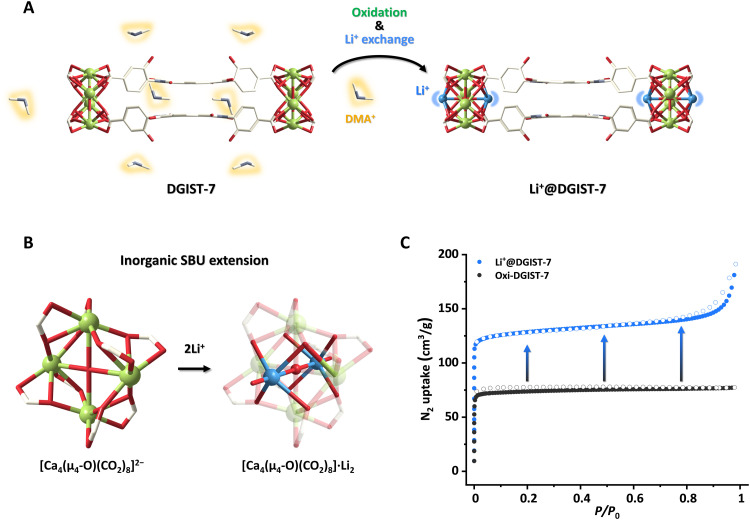
Li^+^ exchange in DGIST-7. (**A**) Scheme depicting Li^+^ exchange in DGIST-7. Solvent molecules coordinated to Li^+^ are omitted for clarity. (**B**) Inorganic SBU extension of DGIST-7 during Li^+^ exchange. (**C**) N_2_ adsorption-desorption isotherms of oxi-DGIST-7 and Li^+^@DGIST-7.

Because ligand reduction to (**L**^2−^)^•−^ and (**L**^2−^)^2−^ promotes its formation, DGIST-7 is presumed to be a stable radical. The oxidative quenching of pristine DGIST-7 was examined by electron paramagnetic resonance (EPR) spectroscopy (Fig. 2A). Samples were prepared by briefly washing the MOF crystals with DMF and then drying under vacuum. The EPR *g* value of 2.003 indicates the presence of a free organic radical, i.e., (**L**^2−^)_2_^•−^ (*29*). Double integration of the EPR peaks revealed that the relative amount of NDI^•−^ grew to 109% over the first 2 hours of air exposure and then gradually decreased to 64% over the next 10 hours and to 3% at 48 hours. Initial peak growth is ascribable to the quenching of EPR-inactive (**L**^2−^)_2_^2−^ to EPR-active (**L**^2−^)_2_^•−^. This quenching is further prolonged in DMF solution; hence, peaks gradually intensified over 3 weeks (Fig. 2B). This unprecedented slow oxidation (stable reduced state) is due to the close proximity of DMA^+^ to (**L**^2−^)_2_^•−^ and (**L**^2−^)_2_^2−^. DMA^+^ is highly disordered and not crystallographically visualizable owing to the coexistence of (**L**^2−^)_2_^•−^ and (**L**^2−^)_2_^2−^ in DGIST-7. When DGIST-7 was prepared in the presence of Cu^2+^ as the (**L**^2−^)_2_^2−^ oxidant, only (**L**^2−^)_2_^•−^ was incorporated into the structure to generate [Ca_4_(μ_4_-O)(**L**_2_^•−^)_2_(DMA^+^)_4_] (denoted as DGIST-7-[Cu^2+^] henceforth). Ordered DMA^+^ in proximity to (**L**^2−^)_2_^•−^ and a metal cluster were observed by SCXRD (Fig. 3A). In addition, a larger NDI centroid-to-centroid distance was observed (3.309 Å) owing to relatively weak interactions associated with (**L**^2−^)_2_^•−^ compared with those of (**L**^2−^)_2_^2−^. The absence of (**L**^2−^)_2_^2−^ in DGIST-7-[Cu^2+^] was further supported by a lack of increase in EPR signal intensity in the initial stage and the observation of comparable peak intensity to the highest observed for DGIST-7 (Fig. 2C). The radical-induced formation of DGIST-7 was further demonstrated by comparing the EPR signal of DGIST-7 with that of 150-DGIST-4 (*61*). While 150-DGIST-4 also contains NDI^•−^-based ligands, its formation is independent of the radical state of the ligand; consequently, only some of the ligands were reduced to radicals during the solvothermal synthesis of 150-DGIST-4. Accordingly, the EPR signal of DGIST-7 is approximately 161 times more intense than that of 150-DGIST-4 (figs. S13 and S14).

### Cyclic voltammetry

DGIST-7 is expected to mainly consist of (**L**^2−^)_2_^•−^ and (**L**^2−^)_2_^2−^. In this regard, pristine and oxidized DGIST-7 (oxi-DGIST-7) were examined by cyclic voltammetry (fig. S18). Oxi-DGIST-7 exhibited two sequential redox peaks at −0.88 and − 1.12 V (versus Ag/AgNO_3_) that correspond to NDI/NDI^•−^ and NDI^•−^/NDI^2−^, respectively (*8*, *40*). Pristine DGIST-7, however, showed no first reduction peak, with only the second peak observed at −1.16 V, albeit not as obviously as that observed for oxi-DGIST-7, which substantiates the presence of (**L**^2−^)_2_^•−^ and (**L**^2−^)_2_^2−^ and the absence of (**L**^2−^)_2_ in pristine DGIST-7.

### Li^+^ exchange in DGIST-7

Despite the porous crystalline structure of DGIST-7, its permanent porosity is not guaranteed because DMA^+^ is present in the pores to balance the anionic [Ca_4_(μ_4_-O)(COO)_8_]^2−^ cluster and NDI^•−^/NDI^2−^ charges (Fig. 3A). The sample was first washed with DMF and ethanol (EtOH) in air to ensure porosity, as the oxidation of (**L**^2−^)_2_^2−^/(**L**^2−^)_2_^•−^ to (**L**^2−^)_2_ releases DMA^+^. The washed oxi-DGIST-7 was then added to a saturated solution of Li^+^ in methanol/EtOH to substitute DMA^+^ with Li^+^, the smallest alkali-metal cation. Li^+^@DGIST-7 was subjected to inductively coupled plasma-optical emission spectrometry (ICP-OES), which revealed a 1:2 ratio of Li to Ca, implying that DMA^+^, which is required to balance the charge of the metal node, had been exchanged for Li^+^. N_2_ adsorption-desorption isotherms acquired at 77 K showed that Li^+^@DGIST-7 has a higher Brunauer-Emmett-Teller surface area (472 m^2^/g) than oxi-DGIST-7 (267 m^2^/g) (Fig. 3C).

### Analyzing the structures of oxi-DGIST-7 and Li^+^@DGIST-7

**Fig. 4.**
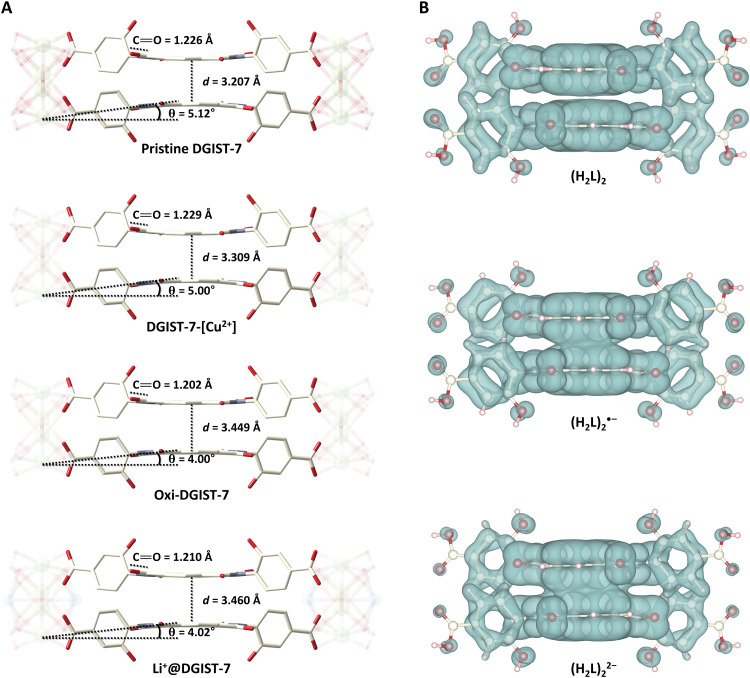
Variations in the interplanar distance between ligand dimers in various DGIST-7 oxidation states. (**A**) Ligand dimers of the four states of DGIST-7 (pristine DGIST-7, DGIST-7-[Cu^2+^], oxi-DGIST-7, and Li^+^@DGIST-7) with their corresponding interplanar distances. (**B**) Charge-density maps of (H_2_**L**)_2_, (H_2_**L**)_2_^•−^, and (H_2_**L**)_2_^2−^.

Despite the lower crystallinity attributable to postsynthetic treatment, the structural transformations that occurred during the oxidation of DGIST-7 and Li^+^ exchange were clearly revealed by SCXRD. First, the distances between the NDI planes in oxi-DGIST-7 (3.449 Å) and Li^+^@DGIST-7 (3.460 Å) were determined to be larger than those in DGIST-7 (3.207 Å) and DGIST-7-[Cu^2+^] (3.309 Å), indicative of weakened π-π interactions resulting from the oxidation of NDI^•−^/NDI^2−^ to NDI (Fig. 4A). As the interplanar distances increased, the warpage angles of **L**^2−^ decreased from 5.12° (DGIST-7) and 5.00° (DGIST-7-[Cu^2+^]) to 4.00° (oxi-DGIST-7) and 4.04° (Li^+^@DGIST-7) (Fig. 4A). The unpaired electron in the NDI^•−^ of DGIST-7 and DGIST-7-[Cu^2+^] was delocalized in the π-conjugated NDI. Thus, the increased single-bond character of the imide carbonyl led to an increase in the bond distances compared to that of the NDI (*62*); the C═O bond distances of DGIST-7 (1.226 Å) and DGIST-7-[Cu^2+^] (1.229 Å) are longer than those of oxi-DGIST-7 (1.202 Å) and Li^+^@DGIST-7 (1.210 Å). The unit cell parameters also change from *a* = *b* = 13.0210(18) Å and *c* = 41.448(8) Å to *a* = *b* = 13.1220(19) Å and *c* = 41.484(8) Å upon the oxidation of DGIST-7 to oxi-DGIST-7, which reflects the interplanar distance change.

Two Li^+^ are incorporated into the [Ca_4_(μ_4_-O)(COO)_8_]^2−^ cluster in Li^+^@DGIST-7, and each Li^+^ is octahedrally coordinated to five oxygen atoms of the cluster and one oxygen atom of a solvent molecule (methanol, EtOH, or water) (Fig. 3B). Since the postsynthetic modification decreases the crystallinity of MOFs, SCXRD is challenging to use in the visualization of inorganic SBU extensions (*63*–*67*).

### Density functional theory analysis

The dimerization energetics of the NDI moieties in various redox states were examined by density functional theory (DFT) calculations. The ligand has three possible redox states: H_2_**L**, H_2_**L**^•−^, and H_2_**L**^2−^; each dimerizes with H_2_**L** to give (H_2_**L**)_2_, (H_2_**L**)_2_^•−^, and (H_2_**L**)_2_^2−^, respectively. The dimerization process is energetically favorable in each case, with formation energies of −21.56, −39.05, and −51.37 kcal/mol, respectively (Table 1) (*45*). Notably, (H_2_**L**)_2_^•−^ and (H_2_**L**)_2_^2−^ have lower formation energies than (H_2_**L**)_2_, which is ascribable to charge delocalization over the cofacially aligned NDI units, as shown in the charge-density maps (Fig. 4B). In addition, the two added electrons in (H_2_**L**)_2_^2−^ form a spin-paired singlet state. The energy gains calculated for the formation of (H_2_**L**)_2_^•−^ and (H_2_**L**)_2_^2−^ support the notion that H_2_**L** dimerizes upon reduction, as observed for DGIST-7. However, a positive formation energy (4.68 kcal/mol) was observed when two H_2_**L**^•−^ molecules dimerize to form (H_2_**L**)_2_^2−^ due to Coulombic repulsion between negatively charged NDI^•−^ units. (H_2_**L**)_2_^2−••^, which is in a metastable spin triplet state, has an even higher formation energy (14.8 kcal/mol) owing to the lack of spin-pairing.

**Table 1.** DFT-calculated formation energies for the neutral, radical-anionic, and dianionic states of H_2_L dimers.

Dimerization				Formation energy
Monomer			Dimer	(kcal/mol)
H_2_**L**	+	H_2_**L**	(H_2_**L**)_2_	−21.56
H_2_**L**	+	H_2_**L**^•−^	(H_2_**L**)_2_^•−^	−39.05
H_2_**L**	+	H_2_**L**^2−^	(H_2_**L**)_2_^2−^	−57.37
H_2_**L**^•−^	+	H_2_**L**^•−^	(H_2_**L**)_2_^2−••^	14.83
			(H_2_**L**)_2_^2−^	4.68

### Generating NDI^•−^ with various stimuli

We assumed that the cofacial arrangement of the NDI dimers in oxi-DGIST-7 is favorable for its reduction to the radical state induced by external stimuli (*21*). Short, repetitive bursts of infrared (IR) radiation (2 W, 30 s, 30×) reduced vacuum-dried oxi-DGIST-7 to the radical state, which exhibited comparable EPR peak intensities to those of the pristine sample while maintaining crystallinity (Fig. 2D and fig. S15). Vacuum-dried oxi-DGIST-7 consists of only two DMF molecules per one ligand, as determined by NMR spectroscopy (fig. S10). In general, irradiation with UV-vis light and the presence of electron donors in the pores are required to produce radical states (*8*, *29*, *68*). Radical formation without an additional electron donor suggests that the electron is transferred within the framework. In addition, various common stimuli, such as heat and UV-vis light, also reduced the NDI of oxi-DGIST-7 (Fig. 2E and figs. S26 to S28). Furthermore, radical-anionic DGIST-7 formed immediately upon exposure to ammonia without any additional stimuli (fig. S29). Notably, DGIST-6 crystals were only slightly reduced under the same conditions, as confirmed by EPR spectroscopy (figs. S30 and S31).

### Photothermal conversion

**Fig. 5.**
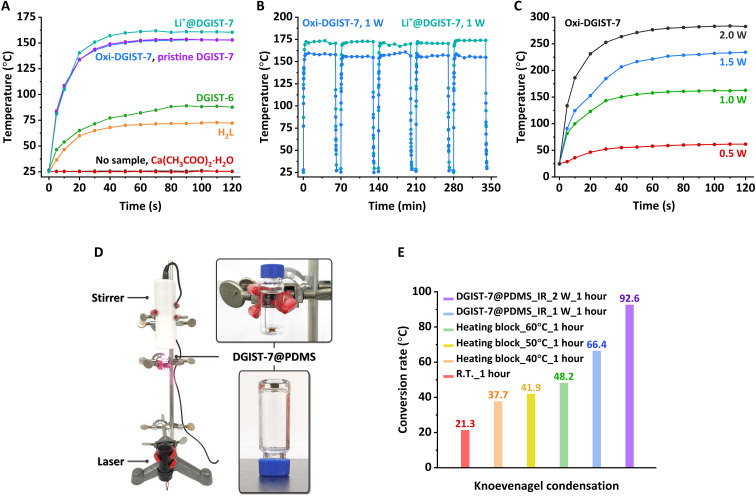
Photothermal conversion of DGIST-7 and its application. (**A**) Comparative photothermal conversion curves for Ca(CH_3_COO)_2_·H_2_O, H_2_**L**, oxi-DGIST-7, pristine DGIST-7, and Li^+^@DGIST-7. (**B**) Repetitive photothermal conversion of oxi-DGIST-7 and Li^+^@DGIST-7. (**C**) Photothermal conversion of oxi-DGIST-7 as a function of the laser intensity. (**D**) Experimental setup for the Knoevenagel condensation with DGIST-7@PDMS. (**E**) Comparison of conversion yields for the Knoevenagel condensation.R.T.- room temperature (25^0^ C).

The NDI bandgap is lowered to the IR region by the addition of electrons, as evidenced by the darkness of the pristine DGIST-7 crystals; this observed IR absorption prompted us to conduct photothermal conversion experiments (*69*–*71*). The temperature readily increased to 150°C within 1 min when pristine crystals were irradiated with a 1-W 808-nm laser (Fig. 5A). Solvent-evacuated oxi-DGIST-7 and Li^+^@DGIST-7 exhibited similar behavior to that of pristine DGIST-7. The efficient photothermal conversion of oxi-DGIST-7 and Li^+^@DGIST-7 is attributable to their conversions into radical-anionic states by IR radiation. In addition, reliable photothermal conversions were achieved for at least 5 cycles of 1-hour irradiation because oxi-DGIST-7 and Li^+^@DGIST-7 were readily reduced by exposure to IR radiation alone; hence, electron donor leaching can be neglected (Fig. 5B) (*72*). Furthermore, the photothermal conversion temperature is proportional to the laser power (Fig. 5C).

This efficient photothermal conversion can be used as a heat source for organic reactions, which was applied to the Knoevenagel condensation of benzaldehyde with malononitrile in DMF as proof of concept (*73*). Oxi-DGIST-7 crystals were loaded into a polydimethylsiloxane (PDMS) mold (DGIST-7@PDMS) to separate the MOF crystals from the reactants (Fig. 5D and fig. S32). The average temperature of the solution was approximately 60°C when irradiated with 2-W 808-nm IR light, while the local temperature of DGIST-7@PDMS increased to 219°C (fig. S34). A conversion yield of 92.6% was observed after 1 hour, which is higher than that (48.2%) obtained by heating the vial on a heating block at 60°C. Localized heating at 219°C presumably accelerates the reaction without solvent boiling.

## DISCUSSION

In conclusion, we reported DGIST-7, a doubly interpenetrated Ca-based MOF. The cofacially oriented NDI dimers in DGIST-7 stabilize NDI^•−^ and NDI^2−^ by through-space electron delocalization, spin-pairing effects, and the presence of the nearby stable counter cation, DMA^+^, as revealed by structural analysis and DFT calculations. Notably, oxi-DGIST-7 was promptly reduced to the radical state by various stimuli. Furthermore, the radical state formed by IR irradiation effectively promoted the photothermal conversion; this process was used to heat an organic reaction. The high crystallinity enabled SCXRD to unambiguously show that Li^+^ is octahedrally coordinated to the anionic [Ca_4_(μ_4_-O)(COO)_8_]^2−^ cluster after counter cation exchange. We successfully crystallographically visualized the structural transitions that occur during the radical quenching of DGIST-7.

## MATERIALS AND METHODS

### Characterization

Powder x-ray diffractometry data were obtained on an Empyrean x-ray diffractometer (Panalytical). SCXRD data were collected using a D8 Venture instrument (Bruker) and synchrotron radiation at the Pohang Accelerator Laboratory Beamline. Synchrotron-based x-rays produced using the PLS-II 2D bending magnet (λ = 0.7 Å) and a Rayonix MX225HS charge-coupled device area detector at 173 K were additionally used to collect diffraction data. Crystallographic data of all samples discussed here were deposited in the Cambridge Structure Database, with deposition codes 2152397 (DGIST-6), 2152398 (pristine DGIST-7), 2152395 (DGIST-7-[Cu^2+^]), 2152399 (oxi-DGIST-7), and 2152396 (Li^+^@DGIST-7). Proton NMR (^1^H-NMR) spectra were recorded on an Avance III Fourier-transform (FT)–NMR 400-MHz spectrometer (Bruker). Chemical shifts are reported in parts per million (ppm) downfield referenced against DMSO-*d_6_* (δ 2.50), the NMR solvent. Elemental analysis was conducted using a Vario MICRO Cube instrument (Elementar). Thermogravimetric analysis was performed on an Auto500 analyzer (TA Instruments) at a heating rate of 5°C/min. IR radiation (808 nm) from an FC-808-5 W Fiber diode-pumped solid state (DPSS) laser (Changchun New Industries Optoelectronics Technology) was used. A xenon lamp (OPS-A500) equipped with a 400-nm long-pass filter and an 800-nm short-pass filter was used for visible (400 to 800 nm) light irradiation. A UV lamp (LF-215.LS, 15 W, 365 nm; UVITEC Cambridge) was adopted for UV light irradiation. EPR spectra were acquired on a JES-FA200 spectrometer (JEOL). ICP-OES was conducted on an iCAP 7400 ICP-OES Duo instrument (Thermo Fisher Scientific). UV-vis spectra were acquired on a UV-2600 spectrometer (Shimadzu), and a CHI 660 D workstation (CH Instruments) was used for all electrochemical experiments. FT-IR spectra were recorded on a Nicolet Continuum infrared spectrometer (Thermo Fisher Scientific).

### DFT calculations

DFT was used to study the energetics of NDI-moiety dimerization, with hydrogen-passivated dimer configurations (H_2_**L**)_2_ in various redox states used as simplified MOF models (*45*). The initial atomic structure was adopted from the π-stack of NDIs in pristine DGIST-7, and the carbons in the carboxylate groups of the ligand dimer were fixed to the corresponding atomic positions in pristine DGIST-7. The rest of the ligand dimer was fully relaxed until atomic forces below 0.01 eV/Å were achieved. DFT total energy calculations were performed using the generalized gradient approximation introduced by Perdew *et al.* (*74*) and the projector-augmented wave (*75*) method as implemented in the Vienna Ab initio Simulation Package (VASP) code (*76*, *77*). An energy cutoff of 400 eV was used for the plane-wave part of the wave function, and van der Waals interactions were included using the DFT-D2 method (*78*). The calculated interplanar distances in (H_2_**L**)_2_, (H_2_**L**)_2_^•−^, and (H_2_**L**)_2_^2−^ are listed in table S4. The added electron in (H_2_**L**)_2_^•−^ (or a pair of electrons in (H_2_**L**)_2_^2−^) participates in the π-bonding of the ligand dimer. The spin density of the EPR-active (H_2_**L**)_2_^•−^ is also plotted in fig. S19, which clearly shows π-bonding character between the ligands.

### Synthesis of H_2_L

Naphthalene-1,4,5,8-tetracarboxylic acid dianhydride (2680 mg, 10 mmol) and 4-amino-3-hydroxybenzoic acid (3093 mg, 20.2 mmol) were added to DMF (40 ml) in a 250-ml two-neck round-bottom flask. The solution was magnetically stirred and refluxed under a stream of N_2_ for 18 hours. After cooling to room temperature, the solution was added dropwise to deionized water (600 ml) and cooled in an ice bath. The obtained precipitate was collected and washed six times (each) with EtOH and acetone and then dried under reduced pressure at 120°C for 12 hours. ^1^H-NMR (400 MHz, DMSO-*d*_6_, ppm) δ: 13.07 (*s*, 2H), 10.18 (*s*, 2H), 8.75 (*s*, 4H), 7.58 (*d*, 2H, and J = 1.8 Hz), 7.55 (*dd*, 2H, and J = 8.1, 1.78 Hz), 7.47 (*dd*, 2H, and J = 8.04, 3.04 Hz).

### Synthesis of DGIST-6 and DGIST-7

Acetic acid (0.25 ml) was added to Ca(CH_3_COO)_2_·H_2_O (14 mg, 0.09 mmol) dissolved in deionized water (0.5 ml). Meanwhile, H_2_**L** (48 mg, 0.09 mmol) was dissolved in DMF (5 ml). The two solutions were mixed in a 16-ml autoclavable vial and then placed in an oven at 150°C. DGIST-6 and DGIST-7 were obtained after 2 and 72 hours, respectively. Elemental analysis calculation (%) for DGIST-6: H, 2.95; C, 57.32; N, 6.47; O, 27.09; Ca, 6.17; found (%): H, 3.28; C, 53.4; N, 6.49; Ca, 5.55. (Ca from ICP-OES analysis). Elemental analysis calculation (%) for DGIST-7: H, 2.03; C, 56.38; N, 4.70; O, 30.18; Ca, 6.72; found (%): H, 3.63; C, 49.75; N, 5.19; Ca, 6.30 (Ca from ICP-OES analysis).

### Synthesis of DGIST-7-[Cu^2+^]

Acetic acid (0.25 ml) was added to Ca(CH_3_COO)_2_∙H_2_O (14 mg, 0.09 mmol) dissolved in deionized water (0.6 ml). Meanwhile, H_2_**L** (48 mg, 0.09 mmol) was dissolved in DMF (4 ml), and CuCl_2_·2H_2_O (3.8 mg, 0.0225 mmol) was dissolved in DMF (1 ml). The three solutions were mixed in a 16-ml autoclavable vial and then placed in an oven at 150°C for 1 day.

### Preparation of oxi-DGIST-7

After washing three times with fresh DMF, the DGIST-7 crystals were briefly immersed in distilled water and then washed nine times with DMF and three times with EtOH. The resulting crystals were soaked in EtOH for 7 days, with the EtOH refreshed daily.

### Preparation of Li^+^@DGIST-7

After washing three times with fresh DMF, the DGIST-7 crystals were briefly immersed in distilled water and then washed nine times with DMF and three times with EtOH. The resulting crystals were soaked in 3.5 M LiCl·H_2_O in a 1:1 (v/v) mixture of MeOH and EtOH for 13 days, with the Li^+^ solution refreshed daily. Last, Li^+^@DGIST-7 was washed several times with fresh EtOH to remove residual (weakly adsorbed) Li^+^. ICP-OES revealed that Li^+^@DGIST-7 has a Li:Ca molar ratio of 0.515:1.000, which is close to the theoretically expected ratio of 0.5:1.0.

### Activating DGIST-7 and Li^+^@DGIST-7

The pristine DGIST-7 crystals were washed three times with fresh DMF, briefly once with distilled water, and then again several times with fresh DMF. The DGIST-7 and Li^+^@DGIST-7 crystals were thoroughly washed with EtOH and cyclohexane for freeze-drying. After as much as possible of the cyclohexane had drained, the MOF crystals were frozen at 253 K and then vacuumed at −10°C for 12 hours to remove cyclohexane. The obtained samples were further vacuumed at 80°C for 12 hours.

### Radical (NDI^•−^) stability testing

The pristine DGIST-7 crystals were washed several times with DMF and dried under vacuum for 18 hours. DGIST-7 crystals (10 mg) were added to a glass tube under inert conditions (Ar). The tube was exposed to ambient atmosphere in the dark, and variations in the amount of NDI^•−^ were monitored by EPR spectroscopy at a power of 1 mW, a modulation width of 0.01 mT, a time constant of 0.03 s, a conversion time of 30 s, a modulation amplitude of one time, and a temperature of 25°C.

### Generating NDI^•−^ with various stimuli

The oxi-DGIST-7 crystals were washed several times with DMF and dried under vacuum for 18 hours. The ratio (1:2.01) of H_2_**L** and DMF in vacuum-dried oxi-DGIST-7 was determined by NMR spectroscopy (fig. S10). DGIST-7 crystals (10 or 30 mg for ammonia exposure experiments) were added to a glass tube under inert conditions (Ar), after which they were exposed to various stimuli, including heat (150°C), IR, UV, visible light, and ammonia. NDI^•−^ formation was monitored by EPR spectroscopy at a power of 1 mW, a modulation width of 0.01 mT, a time constant of 0.03 s, a conversion time of 30 s, a modulation amplitude of 10 times for figs. S26 to S30 and one time for fig. S31, and a temperature of 25°C.

### Fabricating DGIST-7@PDMS

The oxi-DGIST-7 crystals were loaded in a PDMS mold for use as a heat source for an organic reaction without directly interacting the MOF crystals with the reactants. Sylgard 184 silicone elastomer and its curing agent were mixed in a 10:1 weight ratio. Ten drops of the PDMS solution (about 260 mg) were added to a 2-ml vial. A 2-mm-diameter wooden stick was placed at the center of the PDMS solution to create a hole, after which the vial was maintained at room temperature for 6 hours to remove bubbles. In sequence, the vial was then placed in an oven at 60°C for 12 hours to solidify the PDMS mold, and the oxi-DGIST-7 crystals (5 mg) were carefully added into the hole, after which the PDMS solution (four drops, about 100 mg) was added onto the crystals and cured at 60°C for 12 hours. The color of the MOF crystals changed from light yellow to dark brown during curing, indicative of the reduction of oxi-DGIST-7 to DGIST-7.

### Knoevenagel condensation

Benzaldehyde (0.102 ml, 1 mmol) and malononitrile (0.063 ml, 1 mmol) were dissolved in DMF (1.5 ml). Condensation was conducted for 1 hour under six heating conditions: (i) without heating and with heating at (ii) 40°, (iii) 50°, and (iv) 60°C using a heating block, and with heating using (v) 1-W and (vi) 2-W IR laser light. The solutions were magnetically stirred during the reaction. The reaction progress was monitored by NMR spectroscopy, with conversion rates calculated from peak integrals (fig. S35).

## Supplementary Materials

### This PDF file includes:

Supplementary Text Figs. S1 to S35 Tables S1 to S4 References
