# Supplementary material for: π-Stacks of radical-anionic naphthalenediimides in a metal-organic framework
Source: Sci Adv. 2022 Dec 23;8(51):eade1383. doi: 10.1126/sciadv.ade1383 (PMC9788762; doi:10.1126/sciadv.ade1383)
Supplement: Supplementary file 1 — Supplementary Text Figs. S1 to S35 Tables S1 to S4 References [file sciadv.ade1383_sm.pdf]

Science Advances
AAAS

Supplementary Materials for

π-Stacks of radical-anionic naphthalenediimides in a metal-organic framework radical-anionic π-stacks in a MOF

Bongkyeom Kim *et al.*

Corresponding author: Jinhee Park, jinhee@dgist.ac.kr

Sci. Adv. **8**, eade1383 (2022)
DOI: 10.1126/sciadv.ade1383

The PDF file includes:

Supplementary Text
Figs. S1 to S35
Tables S1 to S4
Legends for data S1 to S5
References

Other Supplementary Material for this manuscript includes the following:

Data S1 to S5

#	MOFs	Ligands	Synthesis conditions	Stable conditions	Radical lifetimes	Structural transition analysis	Possible applications	Ref.
1	DGIST-7	*N,N'*-bis(3-hydroxybenzoate)-1,4,5,8-naphthalenediimide	DMF, acetic acid 150 ℃ 72 h	Heat, air, organic solvents (except MeOH)	2 d	O	Photothermal conversion, heat source for chemical reactions	**This work**
2	$[Ca_2(BINDI)(DMF)_4]\cdot 2DMF$	BINDI = *N,N'*-bis(5-isophthalic acid)naphthalenediimide	DMF 100 ℃ 72 h	Heat	2 d	-	Photochromism, photoluminescent	(*79*)
3	Mg-NDI		DMF 90 ℃ 24 h	Heat	8 h	-	Photochromism, solvatochromism, colorimetric and fluorescence sensor of small-sized amine molecules, inkless and erasable printing	(*28*, *29*)
4	Ca-NDI				24 h	-	Inkless and erasable printing	
5	Sr-NDI							
6	FJI-Y1		DMF, HCl 120 ℃ 72 h	Heat, air, organic solvents	Several h	O	Aerobic cross-dehydrogenative coupling	(*30*)
7	FJI-Y2							
8	DGIST-4		DMF, benzoic acid 75 – 150 ℃ 72 h	Heat, air, organic solvents (except MeOH, Act)	2 d	O	Photothermal conversion	(*61*)
9	DGIST-5		DMF, formic acid 85 ℃ 72 h	Heat	-	-	-	
10	$[Zn_3(DPNDI)_2(NMP)_8\cdot 2(PW_{12}O_{40})]_n$	DPNDI = *N,N'*-di(4-pyridyl)-1,4,5,8-naphthalenediimide	NMP/MeCH-NMP interfacial growth at R.T.	Air	Several min	-	Photochromism, photo-controlled tunable luminescence, selective photocatalytic oxidation of benzylic alcohols	(*31*)
11	$[Zn_2(DPNDI)_2(DMA)_4F_3(H_2PW_{12}O_{40})]_n$		DMA/MeCN-MeCN/MeOH interfacial growth at R.T.	-	40 min (Polyoxometalates as guest molecules)	-	Photo-induced accelerating charge-transfer, photochromism	(*32*)
12	PMC-1		Electrocrystallization in DMA 48 h	Heat	- (Electric field)	-	Electron-conductive porous materials	(*55*)
13	csiMOF-6		DMF 80 ℃ 72 h	Heat, organic solvents	16 h (Dimethylaniline soaking)	-	CO_2 reduction using rhenium molecular electrocatalyst	(*33*)
14	$[ZnSiF_6(DPNDI)_2]$		NMP-NMP/EtOH-EtOH interfacial growth at R.T.	Heat	- (Electron-rich naphthalene derivatives as guest molecules)	-	Tunable luminescence, multicolor emission, luminophores	(*34*)
15	$[Zn(DMF)_2(TTFTC)(DPNI)]$	DPNI = DPNDI, TTFTC = (tetrathiafulvalenetetracarboxylate)	DMF 80 ℃ 48 h	-	- (Electric field with electron-rich	-	Conductive and photoactive materials	(*35*)

					guest molecules)			
16	$[Cd(BPDC)(DPNDI)]\cdot 4.5H_2O\cdot DMF$	DPNDI, $BPDCH_2$ = (4,4'-biphenyldicarboxylic acid)	DMF 80 ℃ 72 h	Heat	7-8 h	-	Luminescent, multichromism	(*36*)
17	ZnW-DPNDI−PYI	DPNDI, PYI = Pyrrolidine-2-yl-imidazole	H2O-MeOH (2:1) 120 C 96 h	Heat, air, H_2O	-	-	Heterogeneous photocatalysts	(*37*)
18	CoW-DPNDI-PYI		H_2O-MeOH 120 ℃ 96 h	Air, H_2O	- (Polyoxometalates as guest molecules)	-	Photocatalytic reduction of aryl halides, catalytic coupling of epoxides with CO_2	(*38*)
19	$[Zn(NDI\text{-}ATZ)(DMF)_2]$	NDI-ATZ = *N,N'*-bis(2-5-monohydrate)-1,4,5,8-naphthalenediimide	DMF 80 ℃ 72 h	Heat, air	1 h	-	Photochromism, electrochromism, MOF films	(*68*)
20	$[Na(TauNDI)_{0.5}(H_2O)_2]$	H2TauNDI = *N, N'*-di(ethanesulfonic acid)-1,4,5,8-naphthalenediimide	H_2O-EtOH 120 ℃ 72 h	Heat	-	-	Photochromism, photoswitchable conductance	(*80*)
21	$[Zn_2(DSNDI)]$	DSNDI = *N,N'*-bis(2-hydroxybenzoate)-1,4,5,8-naphthalenediimide	DMF-EtOH-H_2O 90 ℃ 24 h	Heat	- (TTF doping)	-	n-type semiconductor, light-harvesting, photoconducting and semiconducting MOFs	(*39*)
22	Poly-[Zn(AlaNDI)]	AlaNDI *N,N'*-bis(2-Aminopropanoic acid)-1,4,5,8-naphthalenediimide	DMF 120 ℃ 72 h	Heat, EtOH, aniline	10 min	-	Photoluminescence sensing of toxic hydrazine, selective detection of common VOCs	(*81*)
23	AlaNDI-Ca		DMF 120 ℃ 72 h	Heat, chemical	3 min	-	Photodetectors, radiation detectors, solar cells, and chemical sensors	(*82*)
24	[Zr(TMNDI-COO)]	TMNDI = *N,N'*-bis(2,6-dimethyl-4-benzoic acid)-naphthalenediimide	DMF 100 ℃ 48 h	Chemical	- ($CoCp_2$)	-	Strongly photoreducing chromophore, reductive degradation of CH_2Cl_2	(*40*)
25	[Zr(dcphOH-NDI)]	dcphOH-NDI = *N,N'*-bis(3-hydroxybenzoate)-1,4,5,8-naphthalenediimide	DMF 120 ℃ 72 h	Heat, air, H_2O	- (Electric field)	-	MOF-based thin films for reversible electrochromic behavior	(*83*)
26	$[Zn(NO_3)_2(1)]$	1 = N,N'-(Thiazolo[5,4-d]thiazole-2,5-diylbis(4,1-phenylene))-bis(N-(pyridine-4-yl)pyridin-4-amine	MeCN-DMF 120 ℃ 48 h	-	-	-	Iodine adsorption	(*84*)
27	$[Zn_2(BPPTzTz)_2(tdc)_2]$	BPPTzTz = 2,5-Bis(4-(pyridin-4-	DMF 120 ℃ 15 h	Heat	-	-	Through-space intervalence charge transfer	(*41*)

		yl)phenyl)thiazolo[5,4-d]thiazole Tdc = 2,5-thiophene dicarboxylate						
28	$[Zn_4(BDPPTzTz)_2(tdc)_2]$	BDPPTzTz = 2,5-Bis(3,5-dimethyl-4-(pyridine-4-yl)phenyl)thiazolo[5,4-d]- thiazole	DMF 120 ℃ 72 h					
29	$[Zn_2(DPPTzTz)_2(SDC)_2]$	DPPTzTz = 2,5-bis(4-(4-pyridinyl)phenyl)thiazolo[5,4-d]thiazole SDC = selenophene-2,5-dicarboxylate	DMF 120 ℃ 16 h	-	-	-	Through-space intervalence charge transfer	(*42*)
30	$[Cd_2(DPPTzTz)_2(SDC)_2]$							
31	[Cd(BPPTzTz)(tdc)]·2DMF	BPPTzTz, Tdc	DMF 120 ℃ 16 h	Heat	-	-	Through-space intervalence charge transfer	(*43*)
32	Zr-PDI	*N,N'*-di-(4-benzoic acid)-1, 2, 6, 7-tetrachloroperylene-3, 4, 9, 10-tetracarboxylic acid diimide	DMF, acetic acid 90 ℃ 72 h	Heat, H_2O, organic solvents, pH 10, 1 M HCl	1 month (Triethylamine doping)	-	Photothermal conversion	(*72*)
33	NH2-UiO-66 (Zr)/PDI	PTCDA (3,4,9,10-perylenetetracarboxylic dianhydride)	H_2O, TEA, 4 M HCl 60 ℃ 1 h	Heat, H_2O, chemical	-	-	Photocatalytic Cr (VI) reduction, ROS generation, photocatalyst for wastewater purification	(*85*)

Table. S1.

Radical anionic MOFs and their synthesis conditions, stable conditions, radical lifetimes, structural transition analysis, and potential applications

#	MOF	NDI in Pristine state	Possible reduced states of NDI	Stimuli	Ref.
1	DGIST-7	$NDI^{\bullet-}/NDI^{2-}$	$NDI^{\bullet-}/NDI^{2-}$	Heat, IR, Vis, UV, Amine	**This work**
2	$[Ca_2(BINDI)(DMF)_4]\cdot 2DMF$	NDI	$NDI^{\bullet-}$	Vis	(*79*)
3	Mg-NDI	NDI	$NDI^{\bullet-}$	Vis	(*28*, *29*)
4	Ca-NDI	NDI	$NDI^{\bullet-}$	Vis	
5	Sr-NDI	NDI	$NDI^{\bullet-}$	Vis	
6	FJI-Y1	NDI	$NDI^{\bullet+}/NDI^{\bullet-}$	Vis	(*30*)
7	FJI-Y2	NDI	$NDI^{\bullet+}/NDI^{\bullet-}$	Vis	
8	DGIST-4	$NDI/NDI^{\bullet-}$	$NDI^{\bullet-}$	Heat, IR, Vis, UV, X-ray	(*61*)
9	DGIST-5	NDI	-	-	
10	$[Zn_3(DPNDI)_2(NMP)_8\cdot 2(PW_{12}O_{40})]_n$	NDI	$NDI^{\bullet-}$	UV-Vis	(*31*)
11	$[Zn_2(DPNDI)_2(DMA)_4F_3(H_2PW_{12}O_{40})]_n$	NDI	$NDI^{\bullet-}$	UV-Vis with polyoxometalate (e^- donor)	(*32*)
12	PMC-1	$NDI/NDI^{\bullet-}$	-	Electric field	(*55*)
13	csiMOF-6	NDI	$NDI^{\bullet-}$	Vis, Dimethylaniline soaking	(*33*)
14	$[ZnSiF_6(DPNDI)_2]$	NDI	$NDI^{\bullet-}$	Electron rich naphthalene derivatives	(*34*)
15	$[Zn(DMF)_2(TTFTC)(DPNI)]$	$NDI/NDI^{\bullet-}$	-	Electric field with electron rich guest molecules	(*35*)
16	$[Cd(BPDC)(DPNDI)]\cdot 4.5H_2O\cdot DMF$	NDI	$NDI^{\bullet-}$	UV	(*36*)
17	ZnW-DPNDI−PYI	NDI	$NDI^{\bullet-}$	UV-Vis	(*37*)
18	CoW-DPNDI-PYI	NDI	$NDI^{\bullet-}$	Vis with POMs	(*38*)
19	$[Zn(NDI\text{-}ATZ)(DMF)_2]$	NDI	$NDI^{\bullet-}$	Vis	(*68*)
20	$[Na(TauNDI)_{0.5}(H_2O)_2]$	$NDI/NDI^{\bullet-}$	-	UV, UV-Vis	(*80*)
21	$[Zn_2(DSNDI)]$	NDI	$NDI^{\bullet-}$	Tetrathiofuvalene doping	(*39*)
22	Poly-[Zn(AlaNDI)]	NDI	$NDI^{\bullet-}$	UV	(*81*)
23	AlaNDI-Ca	NDI	$NDI^{\bullet-}$	UV	(*82*)
24	[Zr(TMNDI-COO)]	NDI	$NDI^{\bullet-}/NDI^{2-}$	Vis with $CoCp_2$	(*40*)
25	[Zr(dcphOH-NDI)]	NDI	$NDI^{\bullet-}/NDI^{2-}$	Electric filed	(*83*)

Table. S2.
NDI states of pristine and stimuli-exposed MOFs.

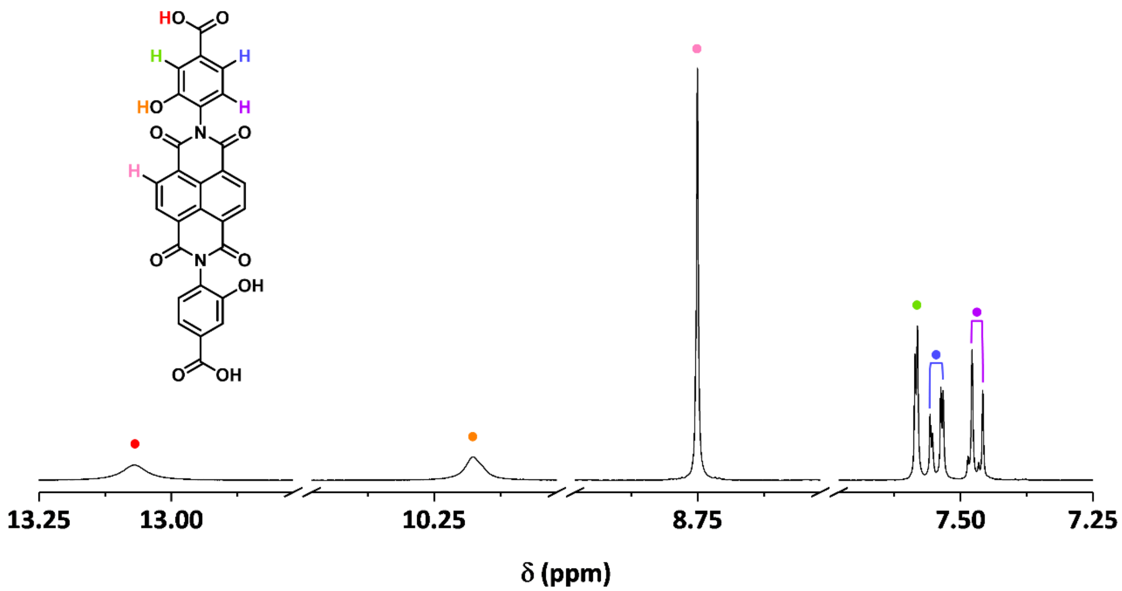

Fig. S1.
^{1}H-NMR spectrum of H_2L.

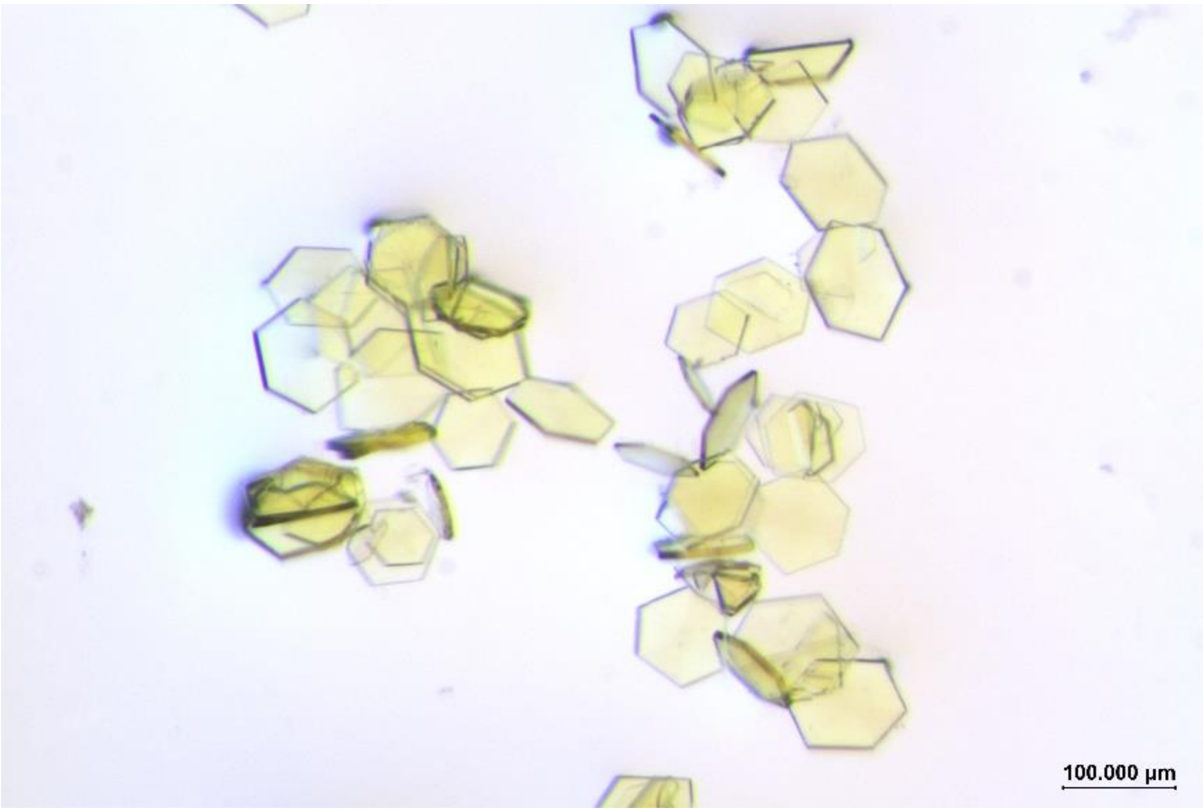

Fig. S2.
Microscope image of the DGIST-6 crystals.

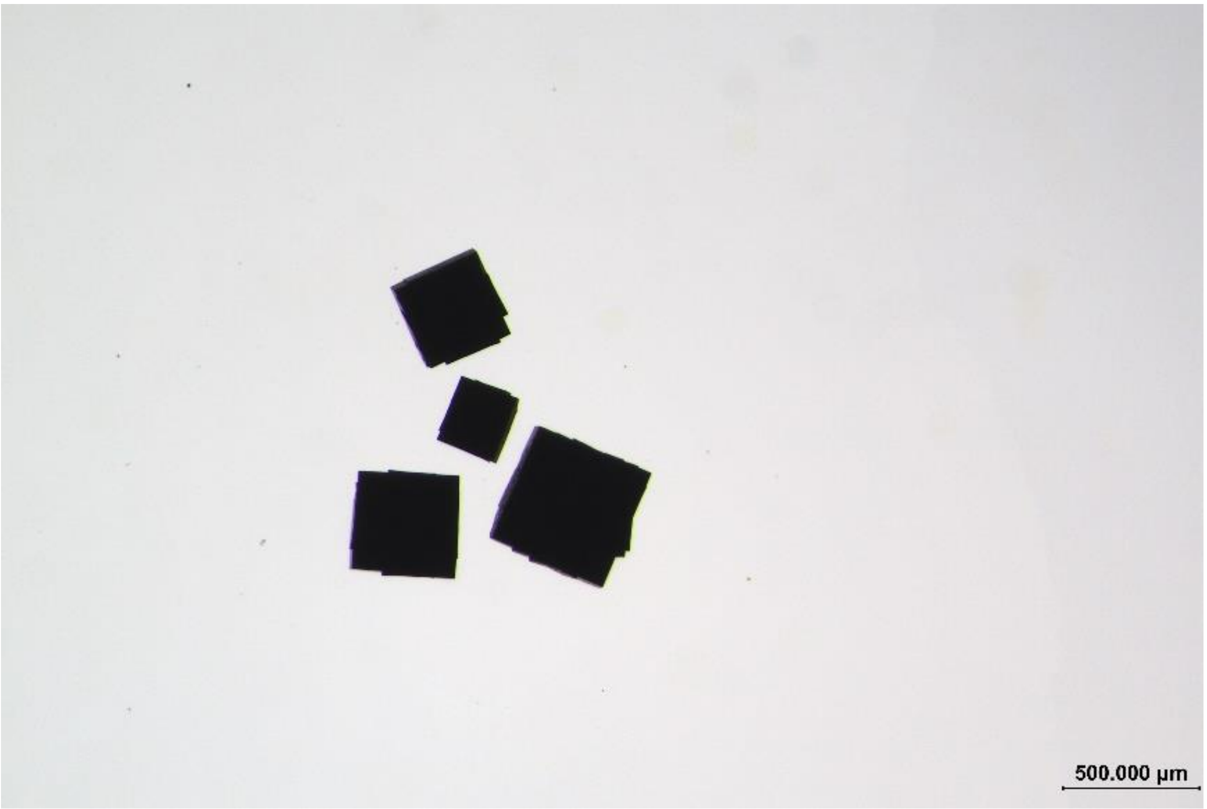

Fig. S3.
Microscope image of the DGIST-7 crystals.

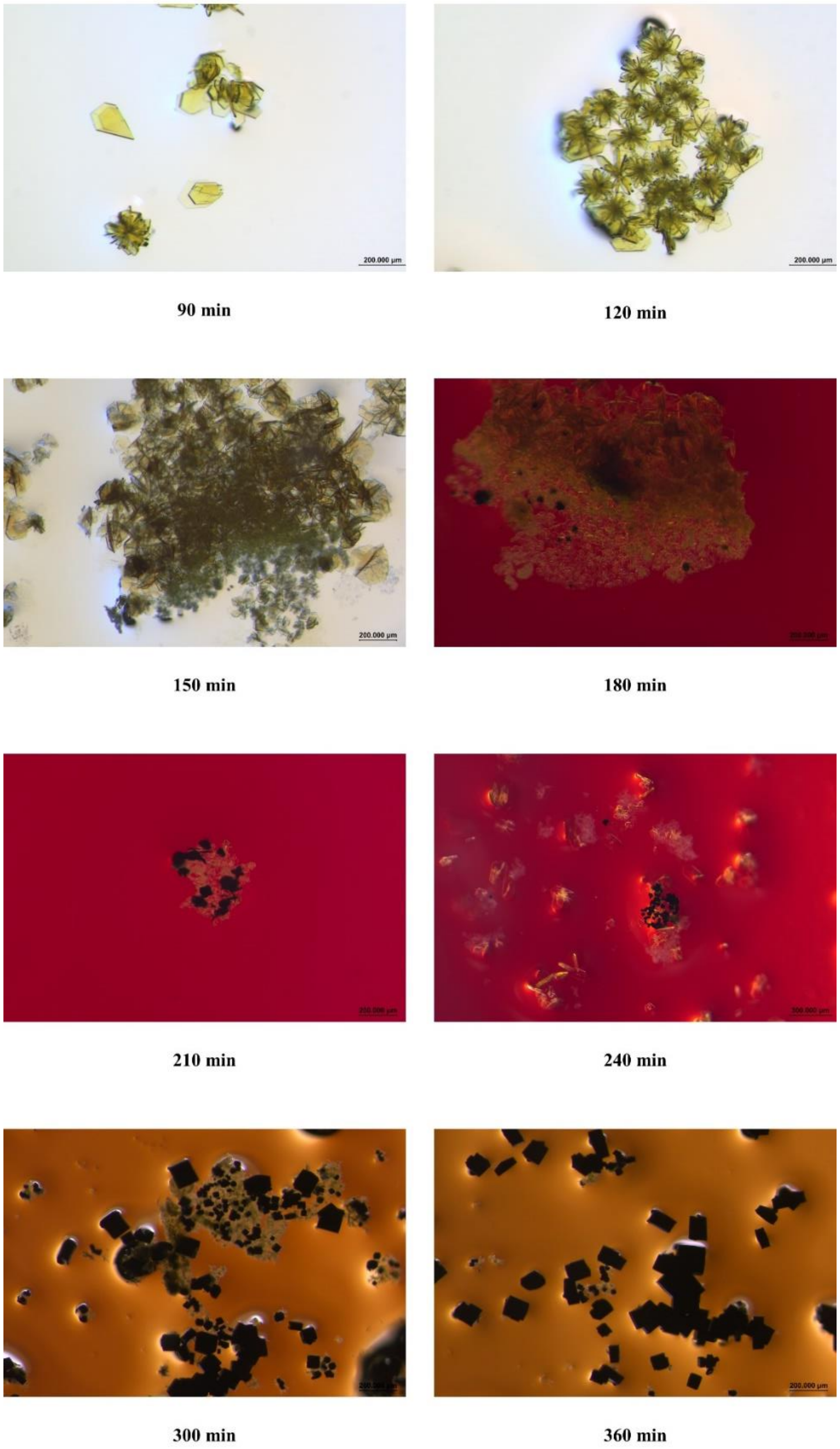

Fig. S4.

Microscope images of the crystals in solution as a function of the reaction time.

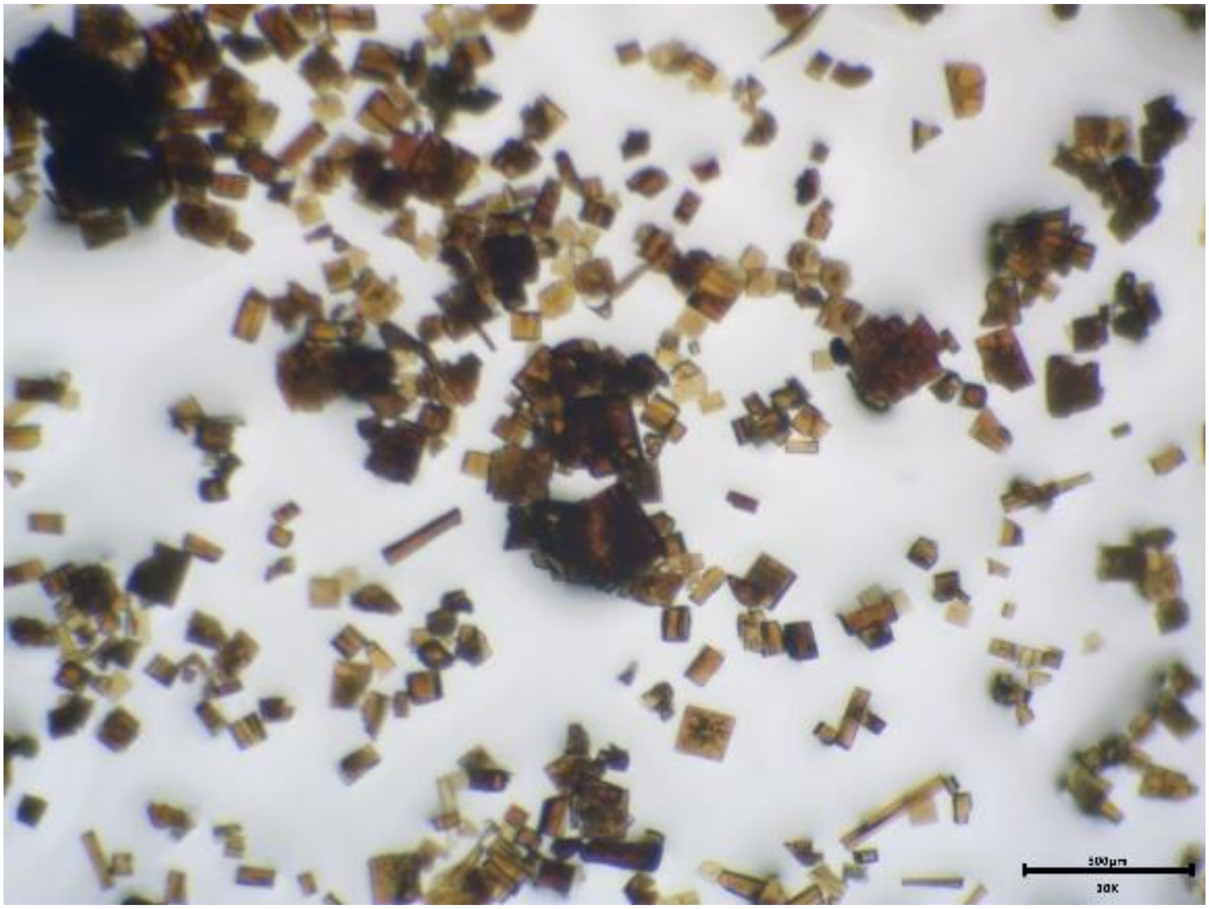

Fig. S5.
Microscope image of the oxi-DGIST-7 crystals.

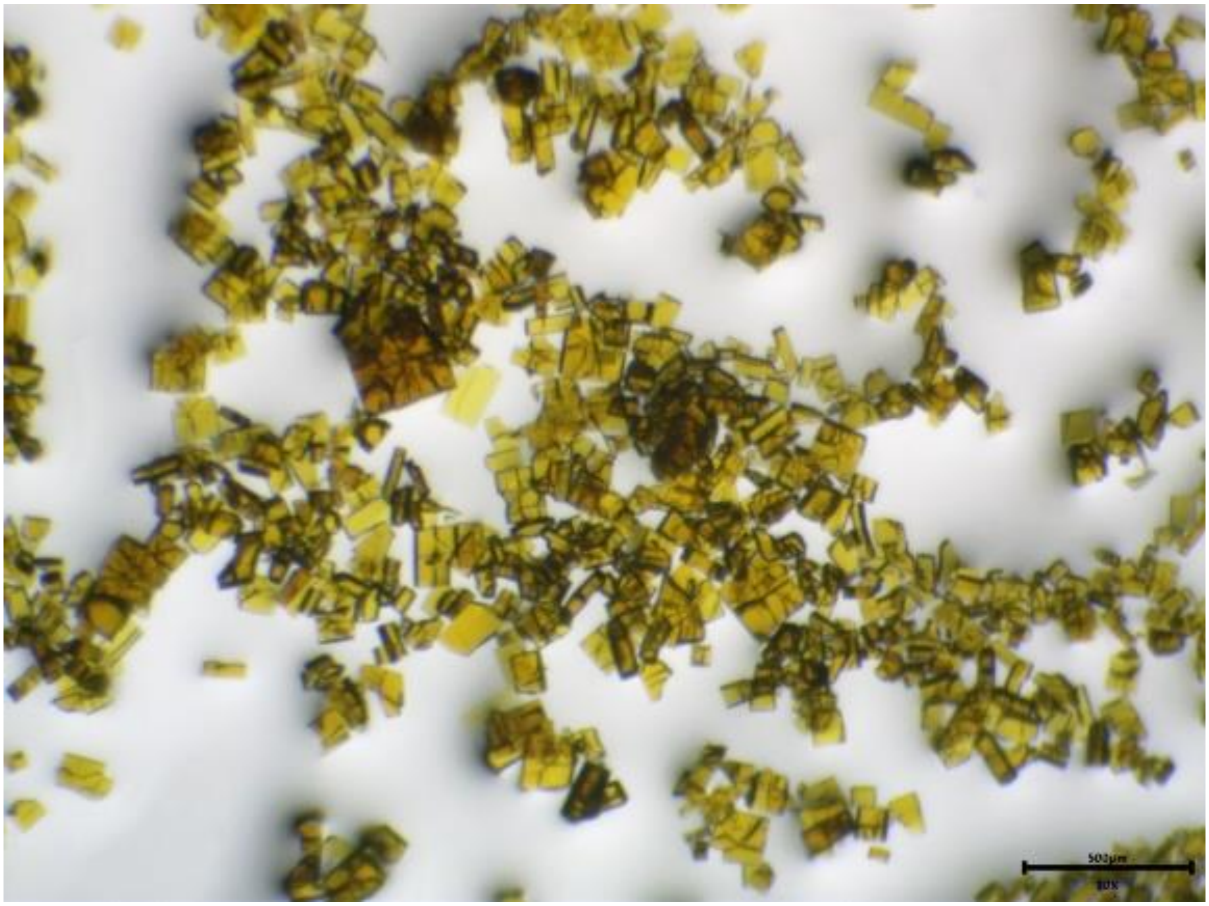

Fig. S6.

Microscope image of the Li^+@DGIST-7 crystals.

Single-crystal X-ray diffractometry

Samples were prepared through the following processes: All crystals were coated with Parabar 10312 (Hampton Research Inc.) and then loaded on a micro-loop. Single-crystal X-ray diffraction (SCXRD) data of pristine DGIST-7, DGIST-7-[Cu^{2+}], oxi-DGIST-7, and Li^{+}@DGIST-7 were collected by the synchrotron-based X-ray source produced from PLS II 2D bending magnet (λ = 0.70000 Å) with a silicon (111) double crystal monochromator and Rayonix MX225HS CCD area detector at 100-223 K. The diffraction frames were recorded by the PAL BL2D-SMDC program (*86*). HKL 3000sm (ver. 717.6) was employed for cell refinement, reduction, and absorption correction (*87*). SCXRD data of DGIST-6 were collected by the Bruker SMART APEX II CCD diffractometer with a monochromator for Mo-K_{α} (λ = 0.71073 Å) incident beam. The data were integrated and scaled by a Bruker-SAINT software. The structures were interpreted ShelXT solution program with the intrinsic phasing method and refined by full-matrix least-squares on F^2 with anisotropic displacement using the SHELXT and Olex2 software package (*88*).
In the structure refinement of DGIST-7-[Cu^{2+}], the space group *P*4/*mmc* was suggested by *Xprep* program. Two disordered counterions (dimethylammonium) were found from the Fourier difference map. Geometry restraints (DFIX) were applied on the counterions during the refinement process. ISOR (isotropic thermal ellipsoid) was applied on atom N4S. The Ca cluster was split as Ca1A-O1A and Ca1B-O1B and the occupancy was refined to be 82.742% for Ca1A and 17.258% for Ca1B, respectively. EADP was applied on O1A and O1B. All non-hydrogen atoms were refined with anisotropic displacement parameters (ADP) during the final cycles. Hydrogen atoms were routinely set to 1.2 × Ueq of the attached atoms of the organic units and 1.5 × Ueq of the attached atoms of the counterions. The strong Q peaks attributed to scattering of disordered solvent molecules were removed using the *SQUEEZE* routine of *PLATON*. Accordingly, the *R*1 was reduced from 14.11% to 9.66%.

The CIF file can be obtained free of charge from the Cambridge Crystallographic Data Centre via www.ccdc.cam.ac.uk/data_request/cif.

Identification code	DGIST-6	DGIST-7	DGIST-7-[Cu^{2+}]	Oxi-DGIST-7	Li^{+}@DGIST-7
CCDC #	2152397	2152398	2152395	2152399	2152396
Empirical formula	$C_{31} H_{21} Ca N_3 O_{12}$	$C_{112} H_{48} Ca_4 N_8 O_{45}$	$C_{120} H_{80} Ca_4 N_{12} O_{45}$	$C_{112} H_{48} Ca_4 N_8 O_{45}$	$C_{112} H_{48} Ca_4 Li_2 N_8 O_{47}$
Formula weight	667.59	2385.9	2570.28	2385.9	2431.78
Temperature (K)	173(2)	100(2)	220(2)	100(2)	220(2)
Wavelength (Å)	0.71073	0.71073	0.71073	0.71073	0.71073
Crystal system	Orthorhombic	Tetragonal	Tetragonal	Tetragonal	Tetragonal
Space group	$P2_12_12_1$	*P4/mcc*	*P4/mcc*	*P4/mcc*	*P4/mcc*
Unit cell dimensions	a = 6.7843(5) Å	a = 13.0210(18) Å	a = 13.3219(10) Å	a = 13.1220(19) Å	a = 13.1840(19) Å
	b = 9.5851(6) Å	b = 13.0210(18) Å	b = 13.3219(10) Å	b = 13.1220(19) Å	b = 13.1840(19) Å
	c = 42.154(3) Å	c = 41.448(8) Å	c = 41.146(2) Å	c = 41.484(8) Å	c = 41.371(8) Å
	α = β = γ = 90°				
Volume ($Å^3$)	2741.2(3)	7027(2)	7302.3(12)	7143(3)	7191(3)
Z	4	2	2	2	2
$d_{calc.}$ (mg/m^3)	1.618	1.128	1.169	1.109	1.123
Absorption coefficient (mm^{-1})	0.308	0.230	0.227	0.227	0.227
F(000)	1376	2432	2648	2432	2476
Theta range for data collection	1.932 to 25.667°	2.512 to 26.372°.	1.821 to 34.176°.	1.836 to 33.965°	1.545 to 34.049°
Index ranges	-8<=h<=8	-11<=h<=10	-20<=h<=20	-19<=h<=19	-19<=h<=19
	-11<=k<=11	-16<=k<=16	-20<=k<=20	-18<=k<=18	-13<=k<=13
	-51<=l<=51	-51<=l<=51	-58<=l<=58	-61<=l<=61	-58<=l<=56
Collected reflections	93260	23946	67022	64741	20733
Independent reflections	5193 [R(int) = 0.1629]	3658 [R(int) = 0.0314]	6424 [R(int) = 0.0850]	6384 [R(int) = 0.0929]	6215 [R(int) = 0.0489]
Completeness to theta = 25.242°	100.00%	99.5%	99.6%	99.8%	99.7%
Max. and min. transmission		1.000 and 0.691	1.000 and 0.789	1.000 and 0.979	1.000 and 0.789
Refinement method	Full-matrix least-squares on F^2				
Data / restraints / parameters	5193 / 0 / 432	3658 / 6 / 203	6424 / 12 / 262	6384 / 6 / 203	6215 / 0 / 203
Goodness-of-fit on F^2	1.095	1.109	1.157	1.008	0.805
Final R indices [I>2sigma(I)]	R1 = 0.0669, wR2 = 0.1539	R1 = 0.0942, wR2 = 0.2930	R1 = 0.0966, wR2 = 0.2982	R1 = 0.0666, wR2 = 0.2246	R1 = 0.0624, wR2 = 0.2125
R indices (all data)	R1 = 0.0788, wR2 = 0.1594	R1 = 0.1034, wR2 = 0.3031	R1 = 0.1256, wR2 = 0.3223	R1 = 0.1157, wR2 = 0.2442	R1 = 0.0960, wR2 = 0.2320
Largest diff. peak and hole ($e.Å^{-3}$)	1.279 and -0.635	1.124 and -0.586	1.797 and -0.567	0.448 and -0.317	0.774 and -0.927

Table. S3.

Crystal data and structure refinements of DGIST-6, DGIST-7, DGIST-7-[Cu^{2+}], oxi-DGIST-7, and Li^{+}@DGIST-7.

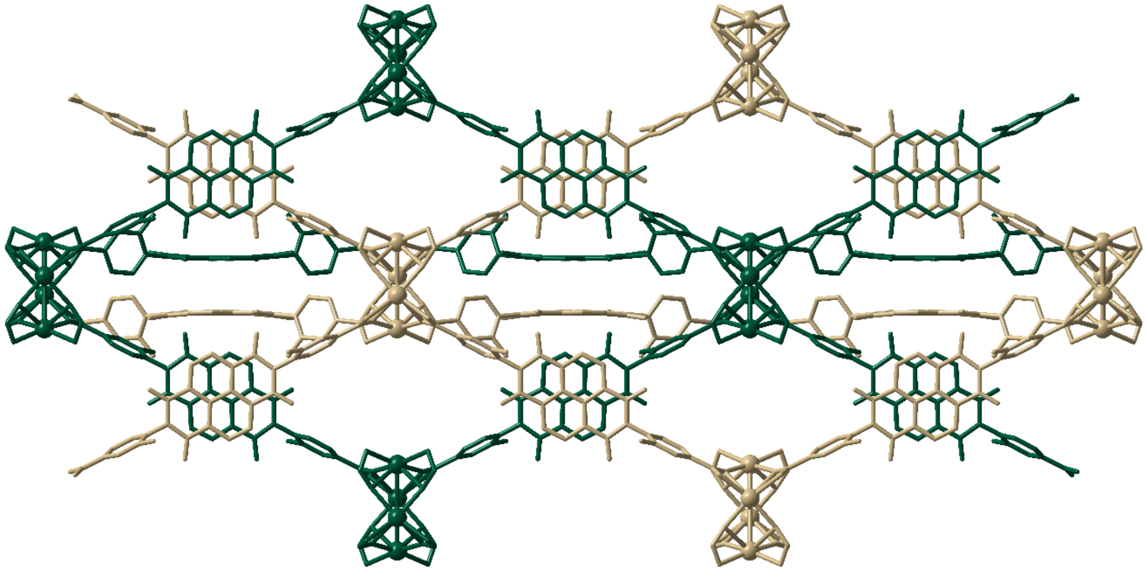

Fig. S7.
Doubly interpenetrated structure of DGIST-7.

NMR analysis of the MOF crystals

The NMR samples were prepared by dissolving 5 mg of the MOF crystals in 0.4 mL of DMSO-d_6 with 1 drop of DCl.

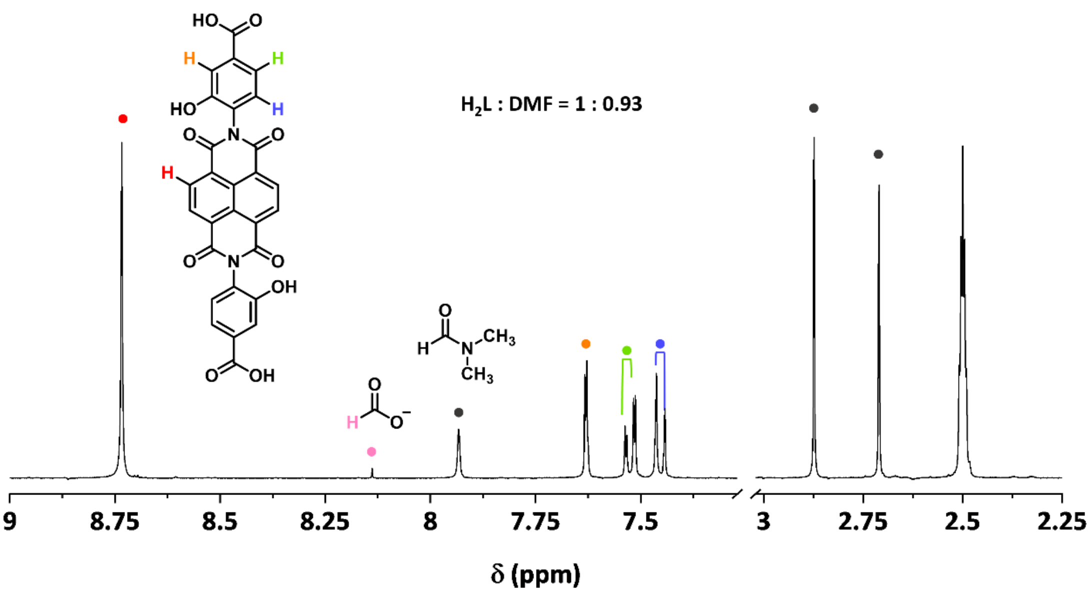

Fig. S8.

NMR spectrum obtained after decomposition of the DGIST-6 crystals.

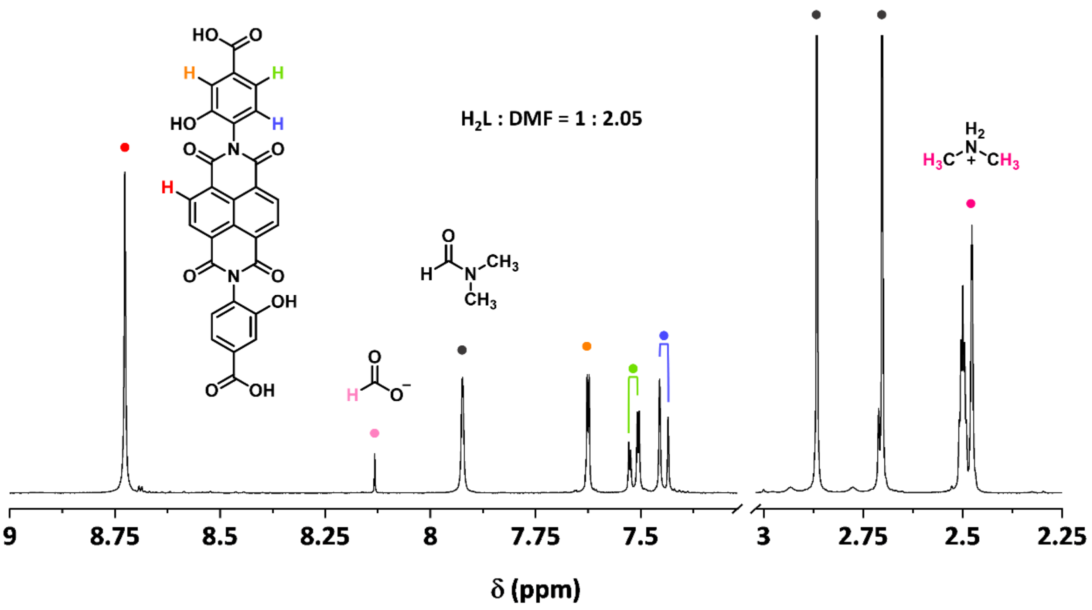

Fig. S9.
NMR spectrum obtained after decomposition of the pristine DGIST-7 crystals.

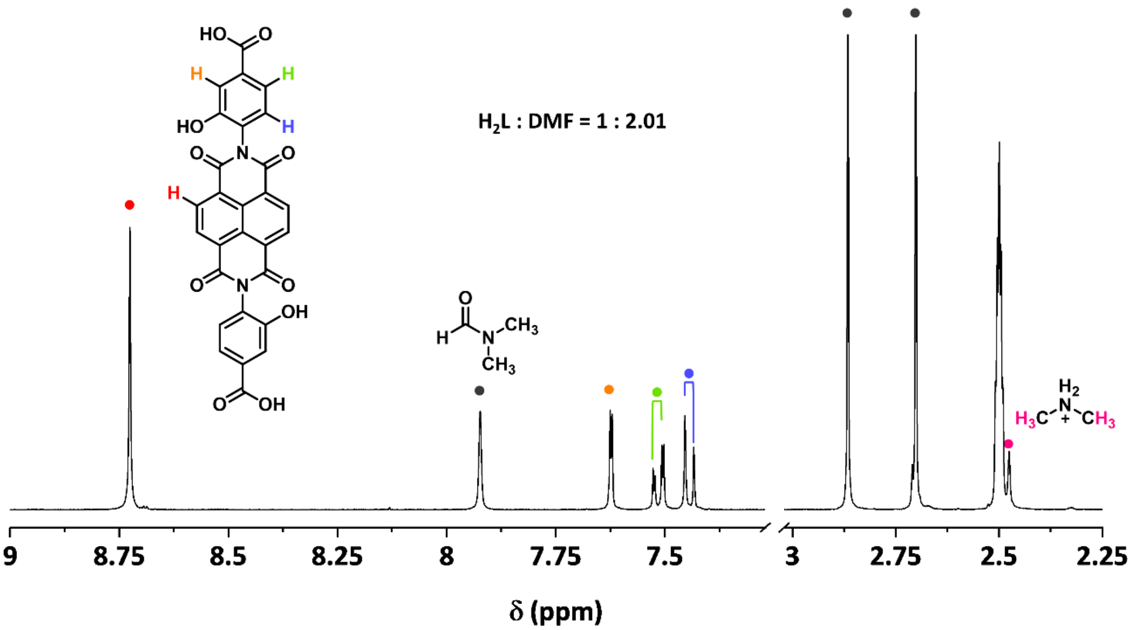

Fig. S10.
NMR spectrum obtained after decomposition of the oxi-DGIST-7 crystals.

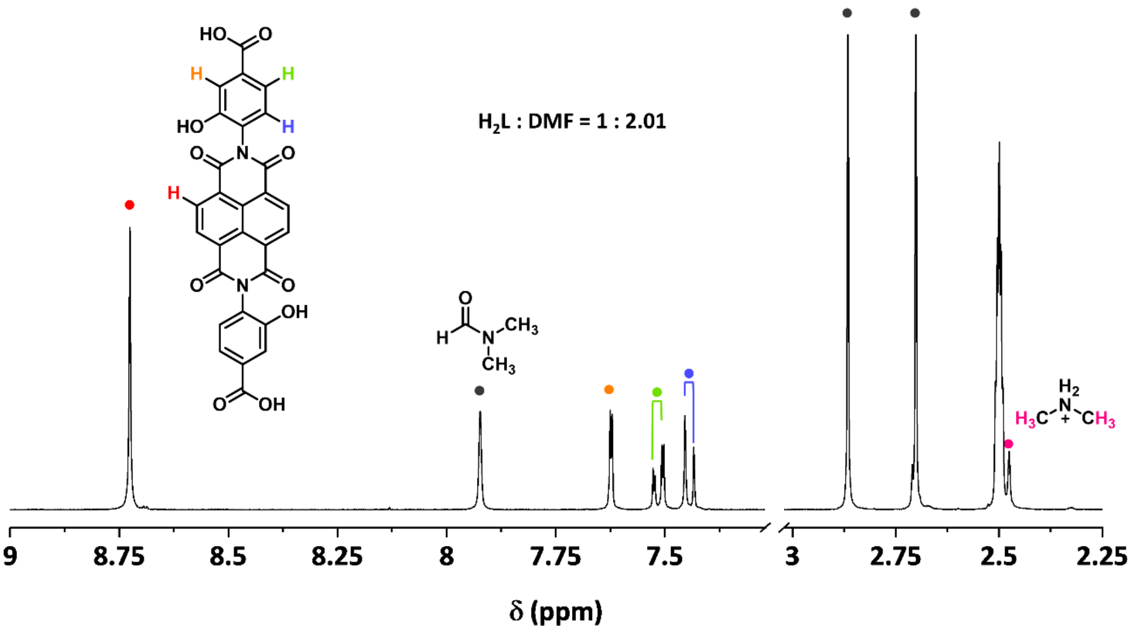

Fig. S11.
NMR spectrum obtained after decomposition of the oxi-DGIST-7 crystals.

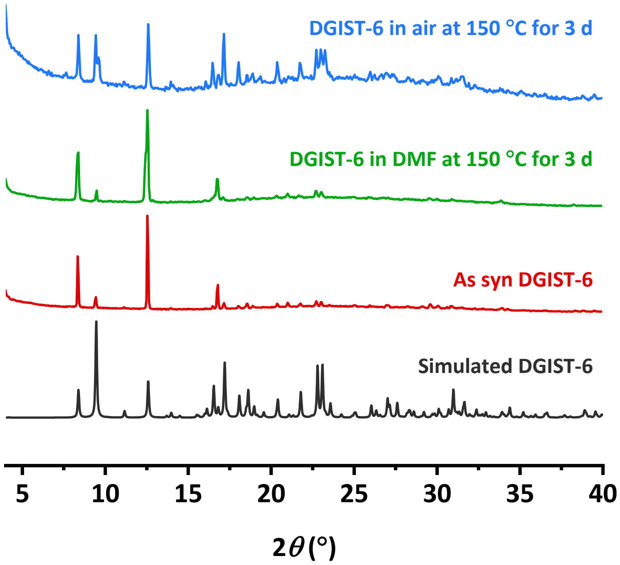

Fig. S12.

Simulated PXRD pattern of DGIST-6 and PXRD patterns of as-synthesized DGIST-6 and DGIST-6 soaked in DMF and exposed in air after 3-day heat treatment at 150 °C.

EPR analysis

Under the inert conditions, 10 mg of pristine DGIST-7 and 18.1 mg of 150-DGIST-4 were transferred to quartz EPR sample tubes, respectively. The EPR spectra were recorded at a power of 1 mW, modulation width of 0.01 mT, time constant 0.03 s, conversion time of 30 s, modulation amplitude of one time, and temperature of 25 ℃.

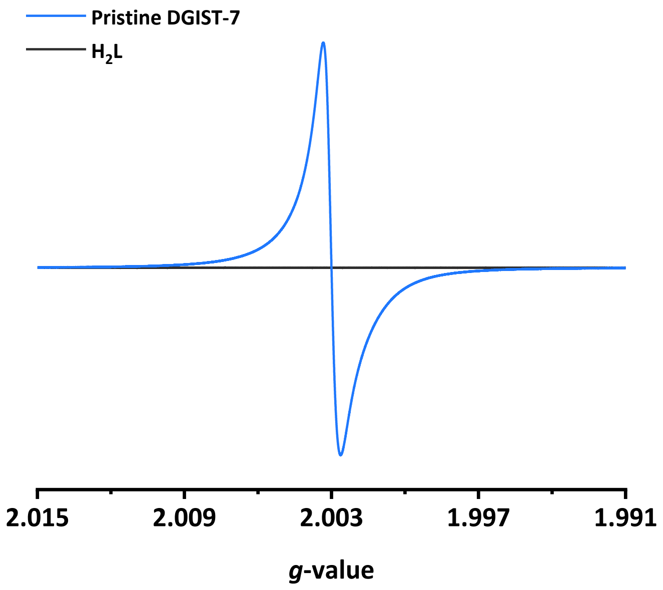

Fig. S13.
EPR spectra of H_2L and pristine DGIST-7. The EPR spectra were recorded at a power of 1 mW, modulation width of 0.01 mT, time constant of 0.03 s, conversion time of 30 s, modulation amplitude of one time, and temperature of 25 °C.

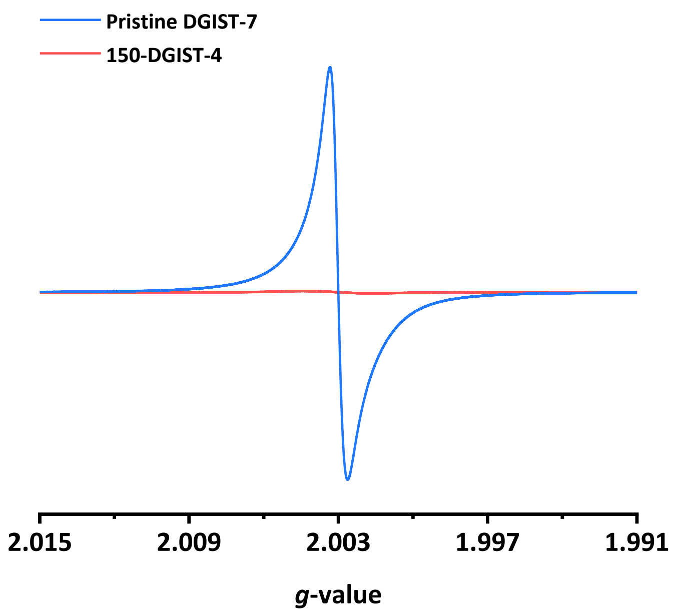

Fig. S14.

EPR spectra of 150-DGIST-4 and pristine DGIST-7. The EPR spectra were recorded at a power of 1 mW, modulation width of 0.01 mT, time constant of 0.03 s, conversion time of 30 s, modulation amplitude of one time, and temperature of 25 °C.

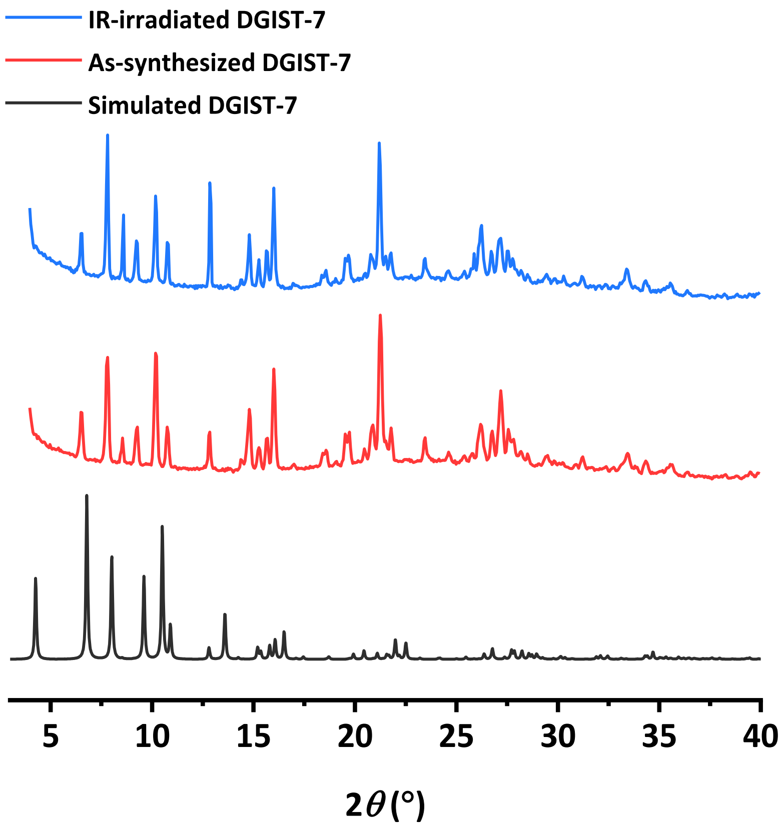

Fig. S15.
PXRD patterns of IR-irradiated DGIST-7 (2 W, 30 s for 30 times).

Solvent stability test

DGIST-6 and DGIST-7, briefly washed with DMF, were soaked in H_2O, MeOH, EtOH, acetone, THF, DMF, DCM, chloroform, and cyclohexane for 1 day. The crystallinity of each sample was analyzed by the PXRD.

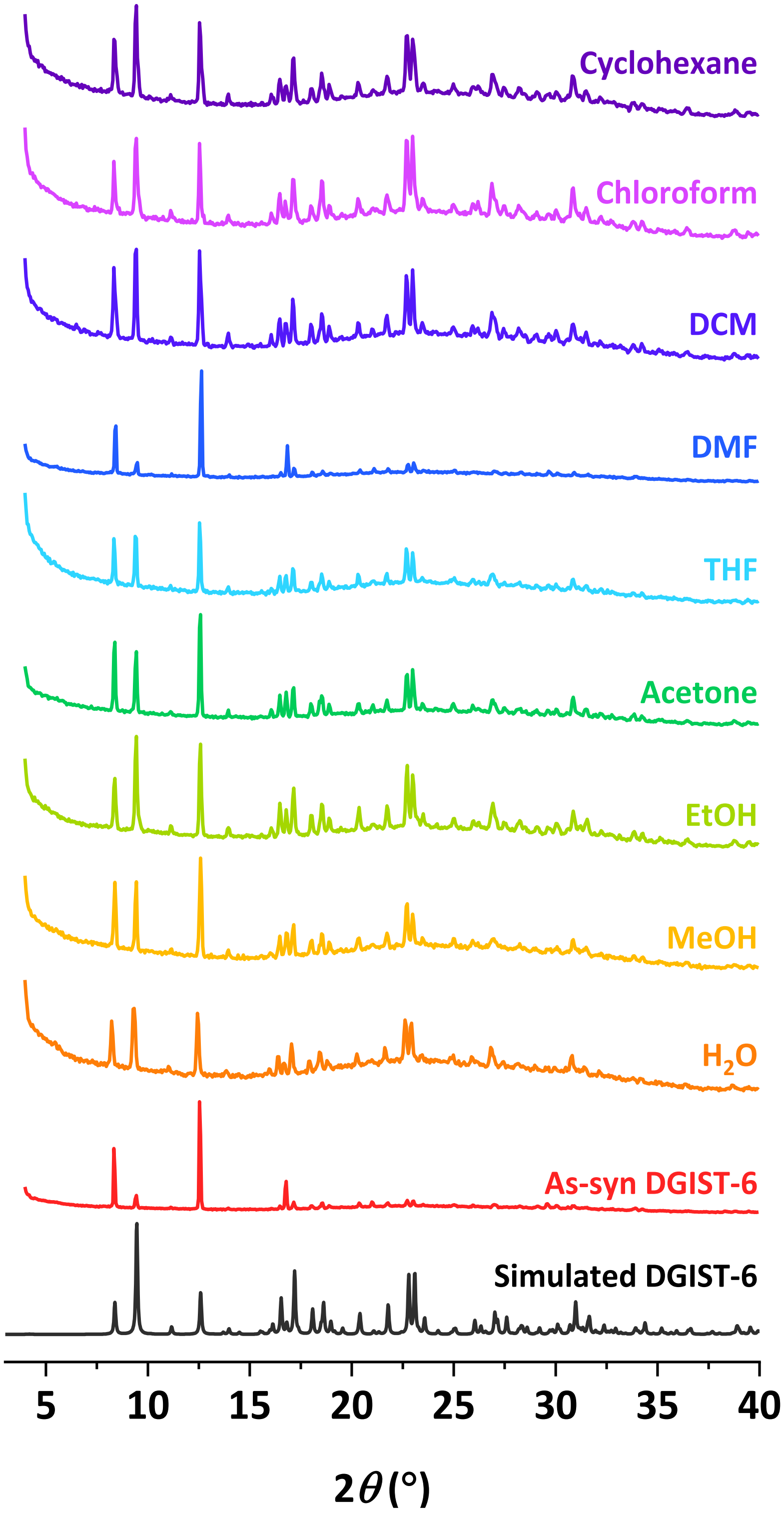

Fig. S16.

PXRD patterns of DGIST-6, obtained after soaking in various solvents.

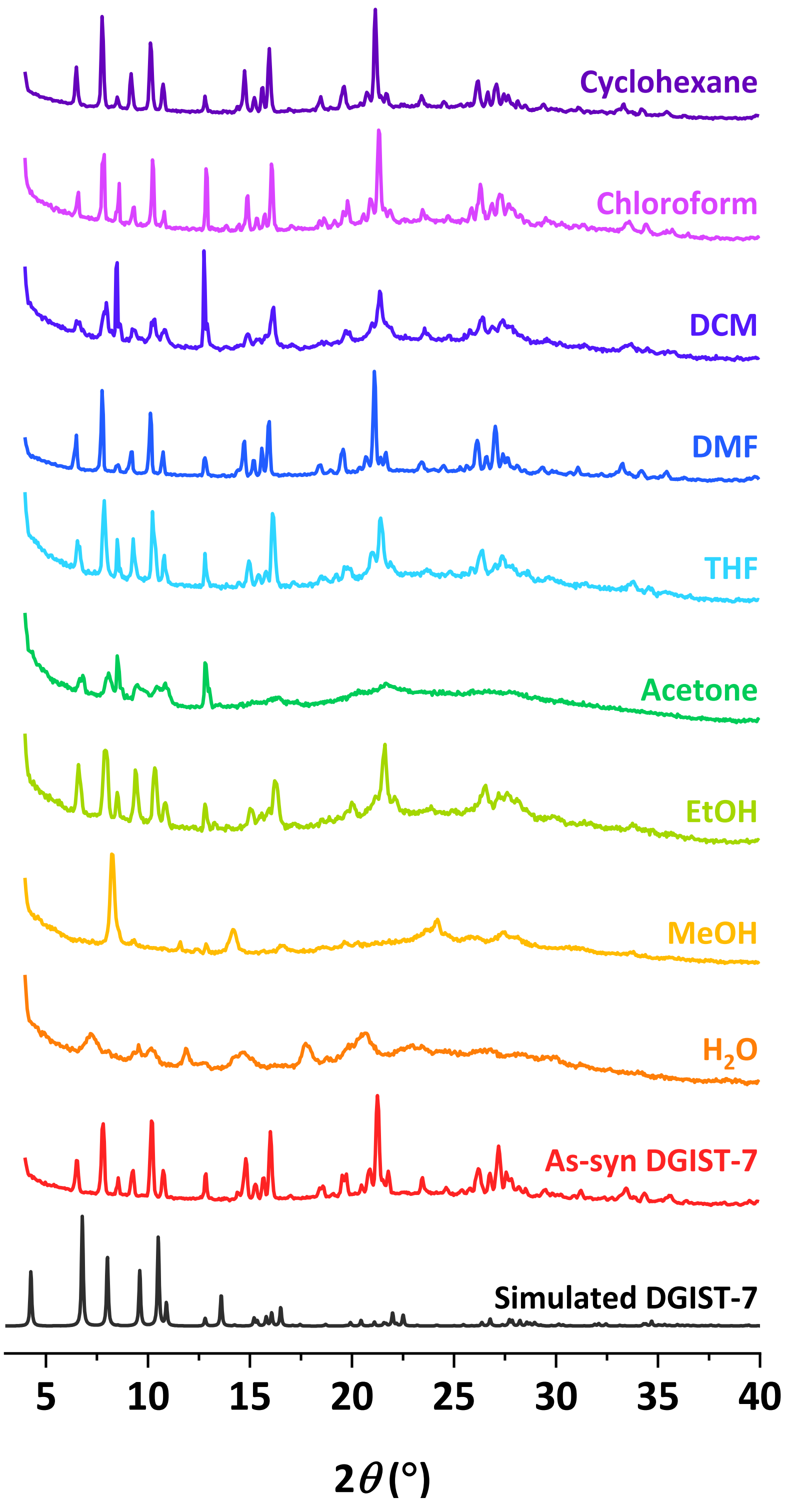

Fig. S17.
PXRD patterns of DGIST-7, obtained after soaking in various solvents.

Cyclic voltammetry

The cyclic voltammogram (CV) measurements were conducted in a three-electrode cell consisting of a Pt mesh counter electrode, a Ag/$AgNO_3$ (10 mM) reference electrode, and the MOFs on an ITO substrate (1.5 cm x 1.5 cm) as a working electrode. The solution of 0.1 M nBu_4NPF_4 in acetonitrile was used as the supporting electrolyte. The CV curves were collected at the scan rate of 0.1 Vs^{-1}.

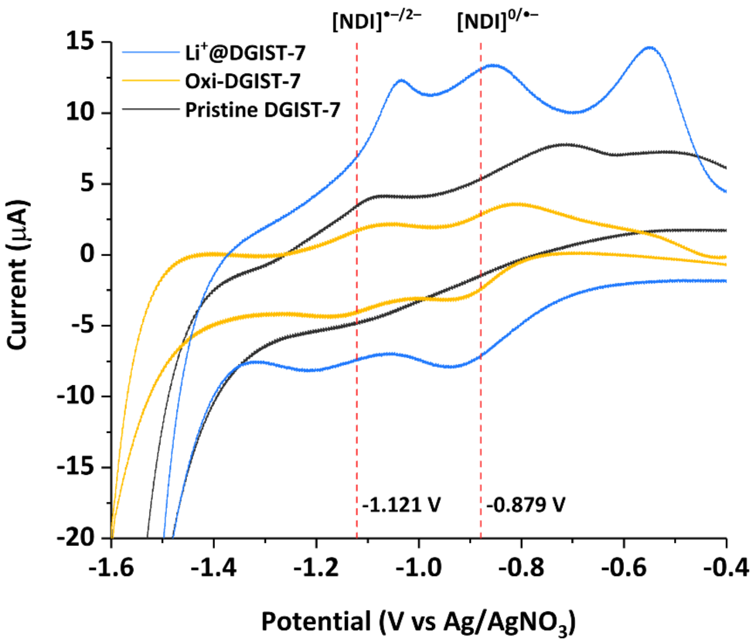

Fig. S18.

Cyclic voltammograms of pristine DGIST-7, oxi-DGIST-7, and Li^{+}@DGIST-7.

Species	Calculated interplanar distance (Å)
$(H_2\mathbf{L})_2$	3.366
$(H_2\mathbf{L})_2^{\bullet-}$	3.204
$(H_2\mathbf{L})_2^{2-}$	3.126

Table. S4.
Calculated interplanar distances of $(H_2L)_2$, $(H_2L)_2^{\bullet-}$, and $(H_2L)_2^{2-}$.

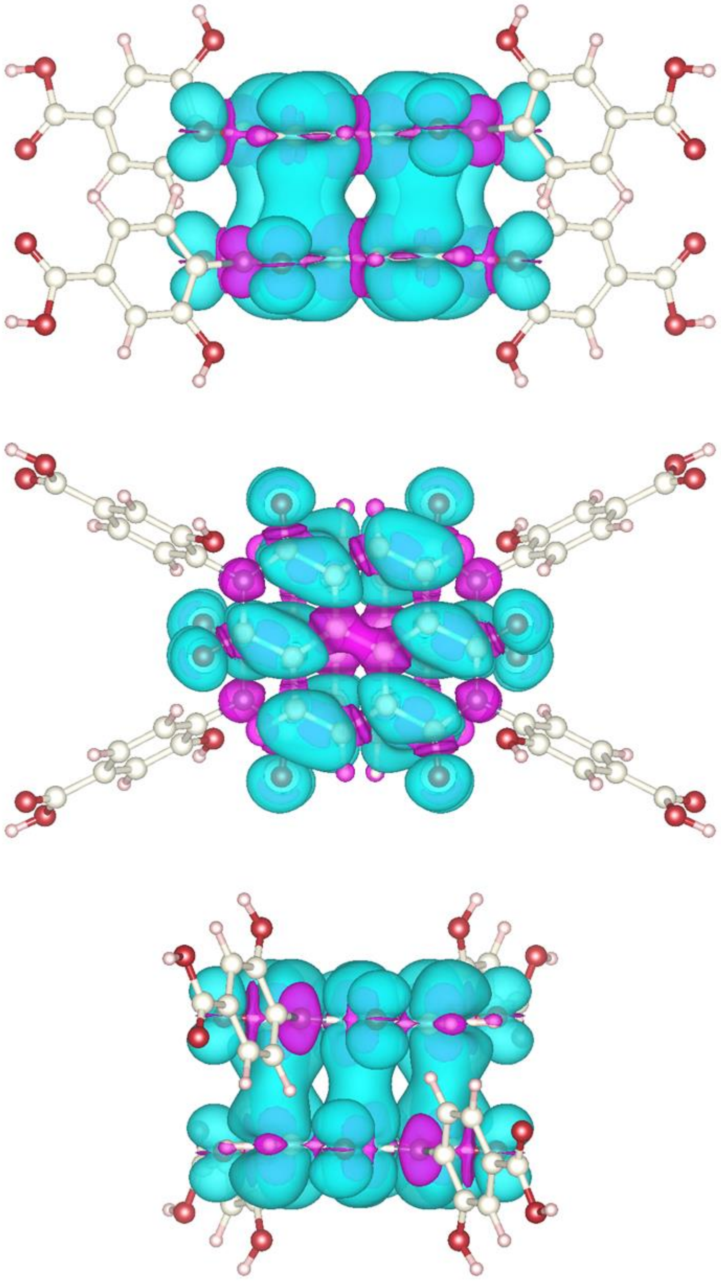

Fig. S19.

Spin density plots of $(H_2L)_2^{\bullet-}$ in different views (blue: spin-up state, purple: spin-down state).

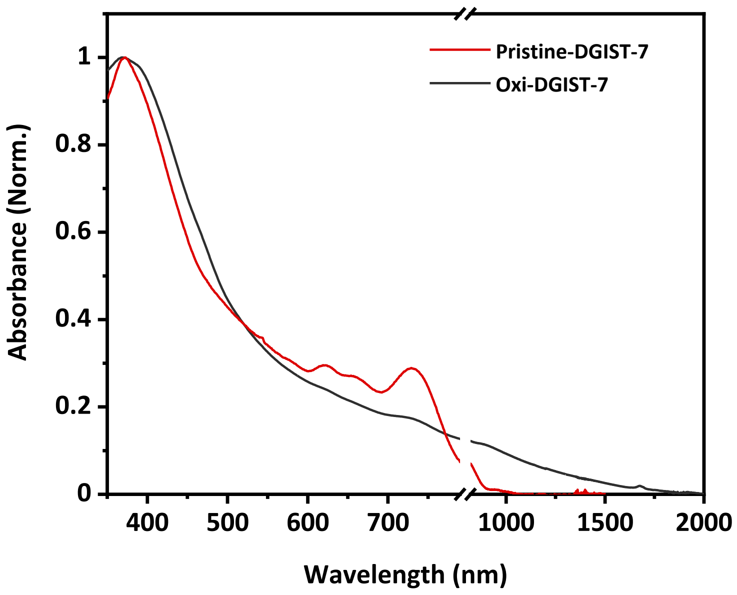

Fig. S20.
UV-Vis-NIR absorption spectra of pristine DGIST-7 and oxi-DGIST-7.

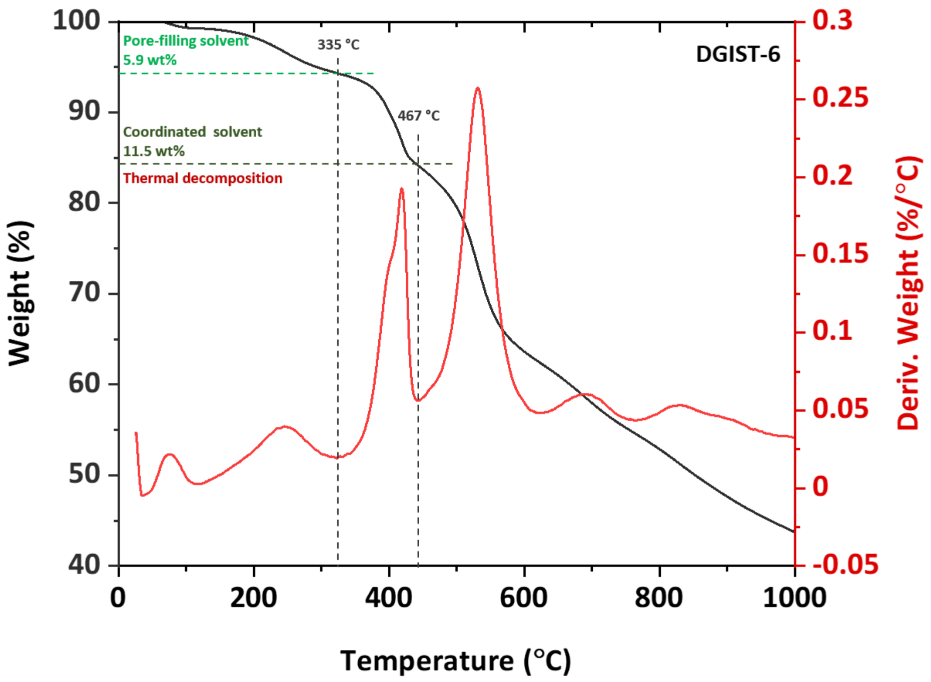

Fig. S21.

Thermogravimetric analysis curve of DGIST-6.

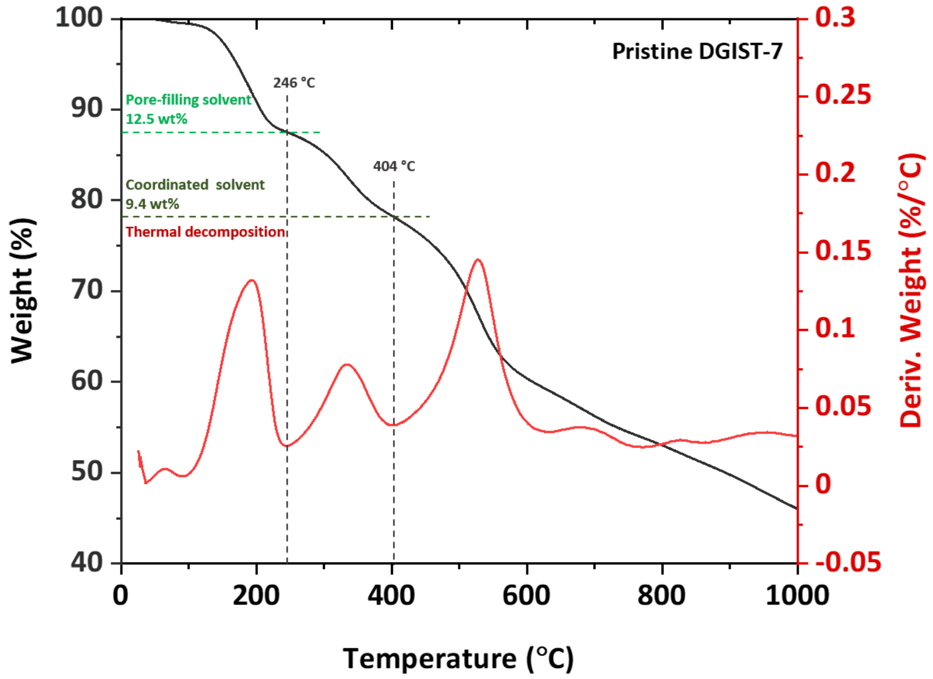

Fig. S22.

Thermogravimetric analysis curve of pristine DGIST-7.

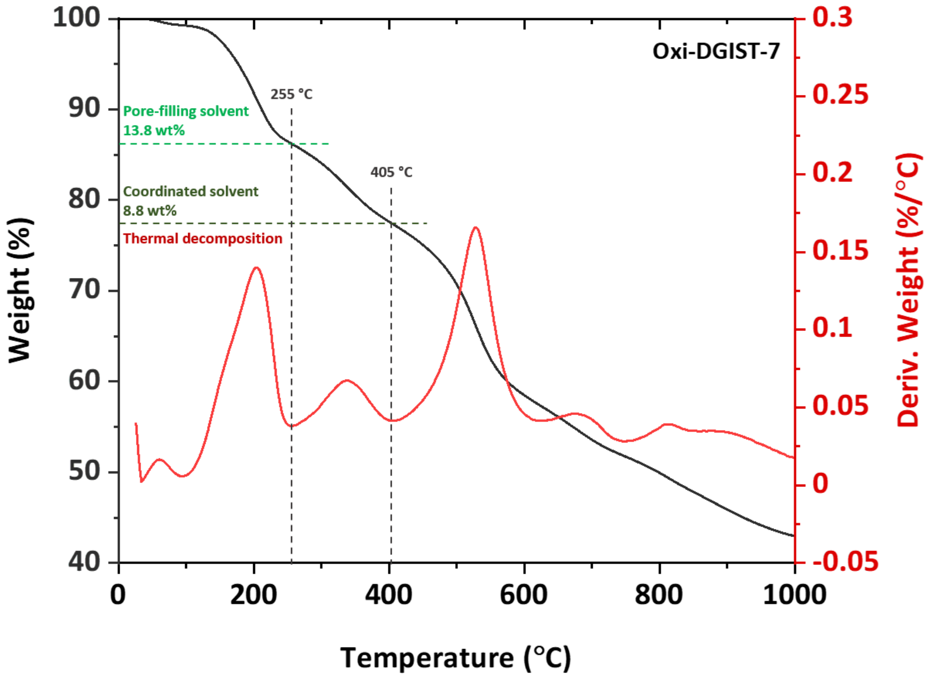

Fig. S23.

Thermogravimetric analysis curve of oxi-DGIST-7.

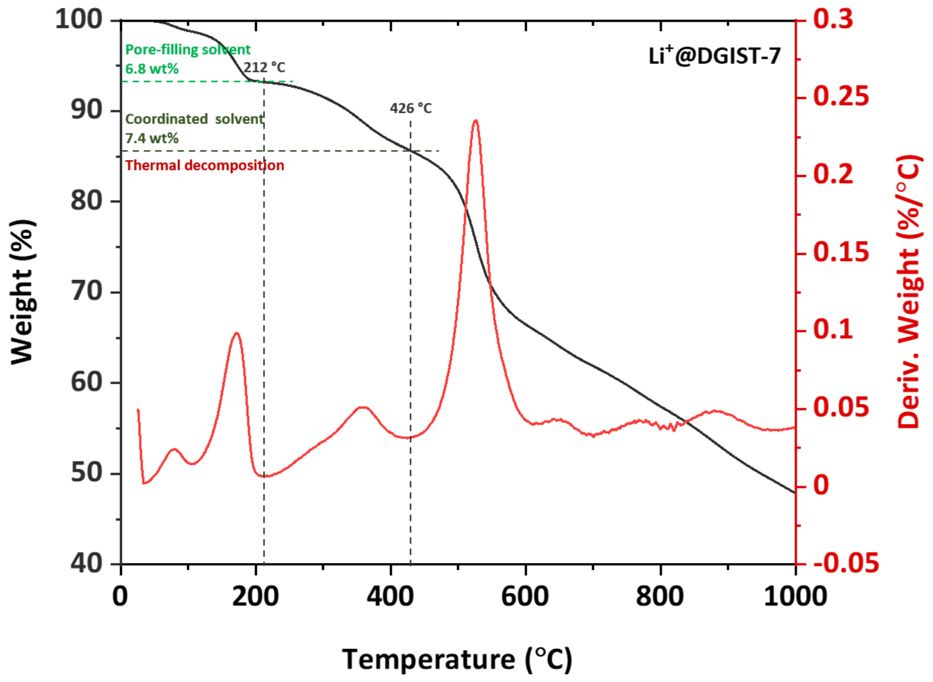

Fig. S24.

Thermogravimetric analysis curve of Li^{+}@DGIST-7.

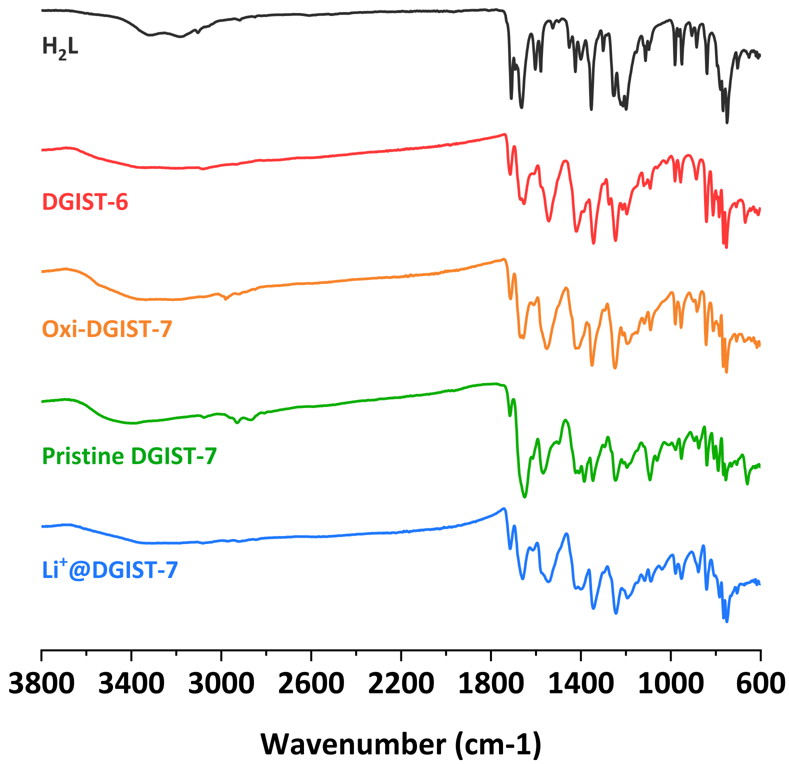

Fig. S25.

FT-IR spectra of H_2L, DGIST-6, oxi-DGIST-7, pristine DGIST-7, and Li^+@DGIST-7.

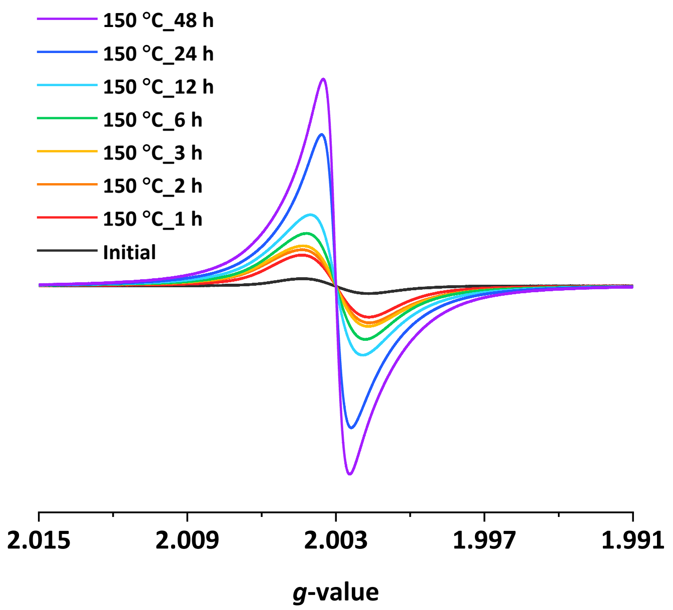

Fig. S26.
EPR spectra of oxi-DGIST-7 after the heat treatment at 150 °C.

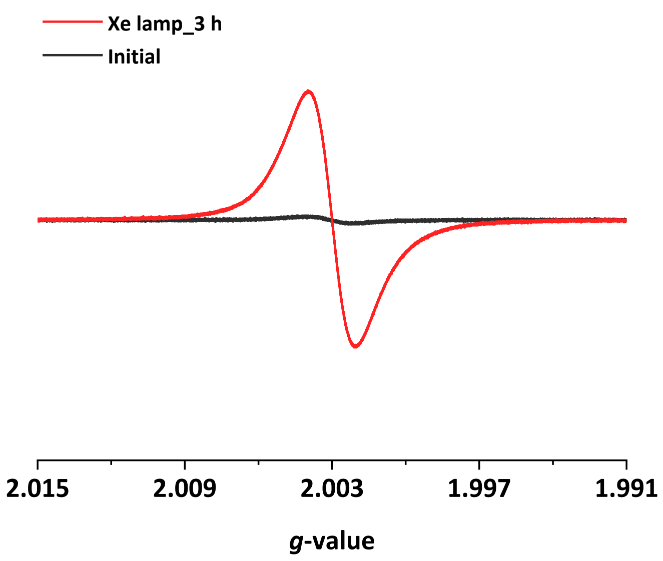

Fig. S27.
EPR spectra of oxi-DGIST-7 before and after visible light irradiation.

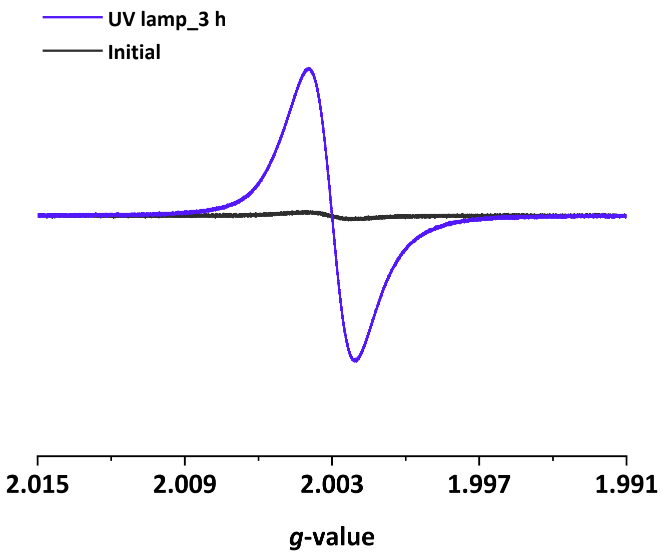

Fig. S28.
EPR spectra of oxi-DGIST-7 before and after UV irradiation.

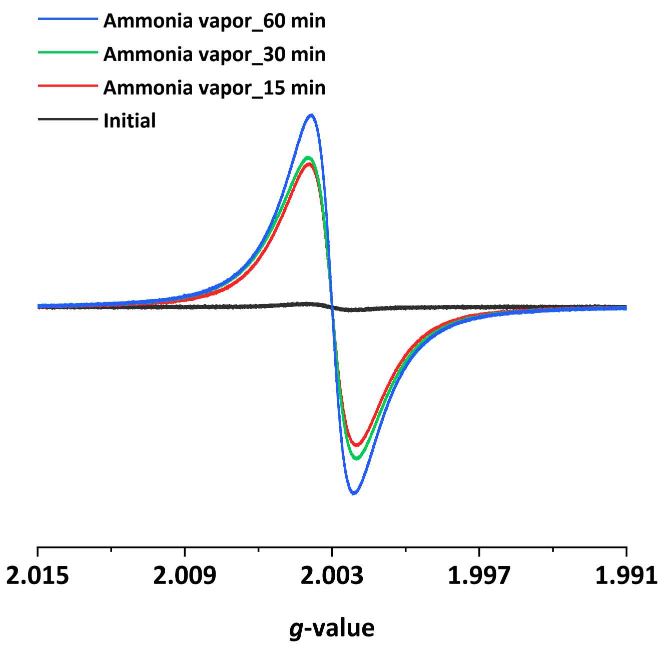

Fig. S29.

EPR spectra of oxi-DGIST-7 exposed to ammonia vapor. Under Ar conditions, 30 mg of DGIST-7 was added in a 4 mL vial without a cap. 2 drops of NH_3 were added to 20 mL of the autoclavable vial and then the capless 4 mL vial with DGIST-7 was placed inside of the 20 mL vial.

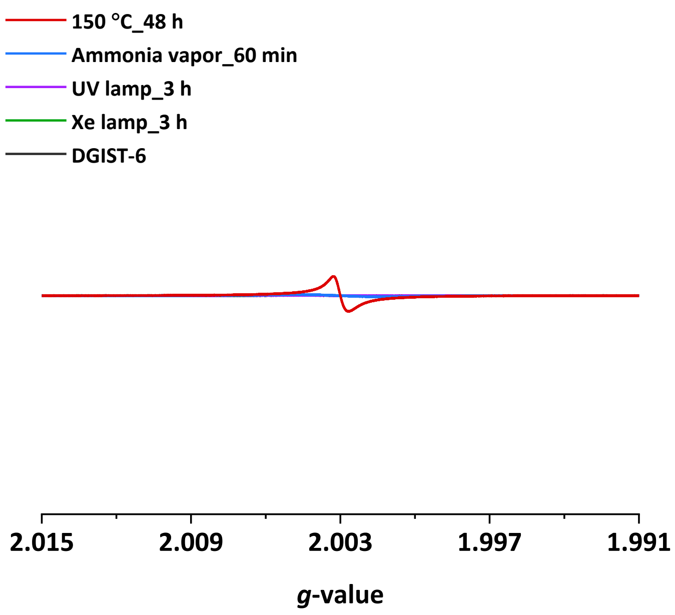

Fig. S30.
EPR spectra of DGIST-6 reduced by the various stimuli (UV, visible light, heat, and ammonia).

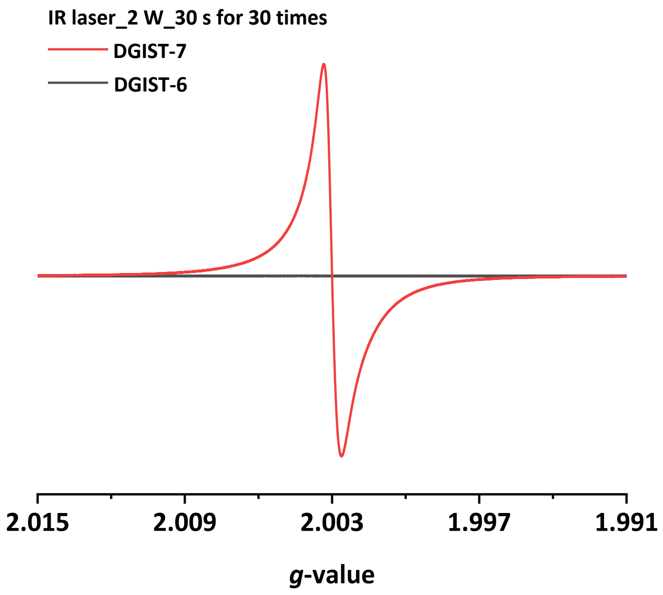

Fig. S31.

EPR spectra of DGIST-6 and DGIST-7 after the repetitive IR laser irradiation (2 W, 30 s for 30 times).

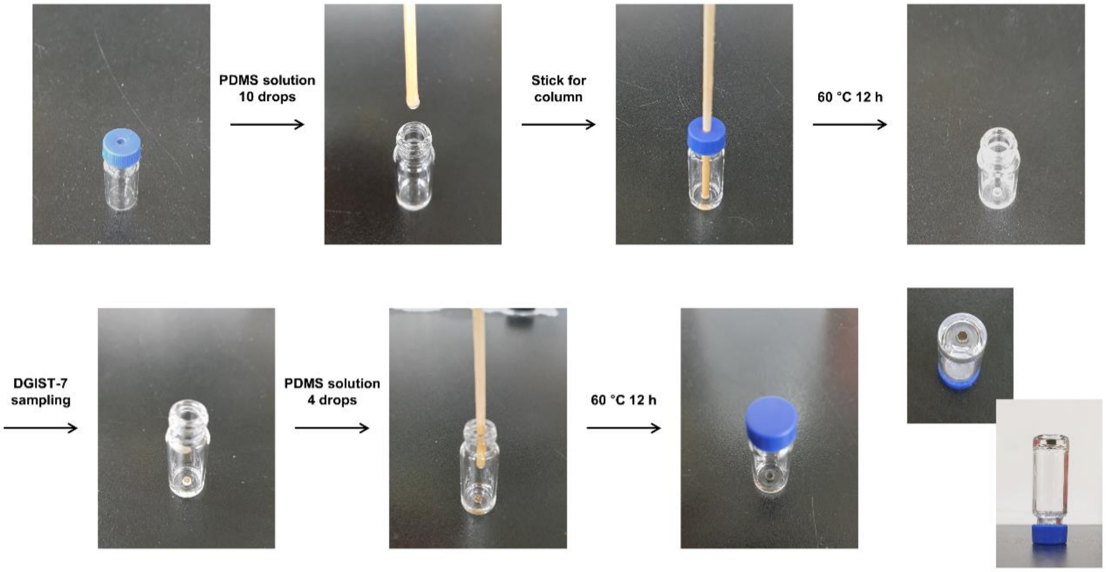

Fig. S32.
The fabrication process of DGIST-7@PDMS in the vial.

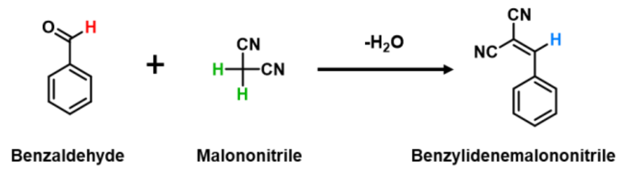

Fig. S33.

Scheme of Knoevenagel condensation.

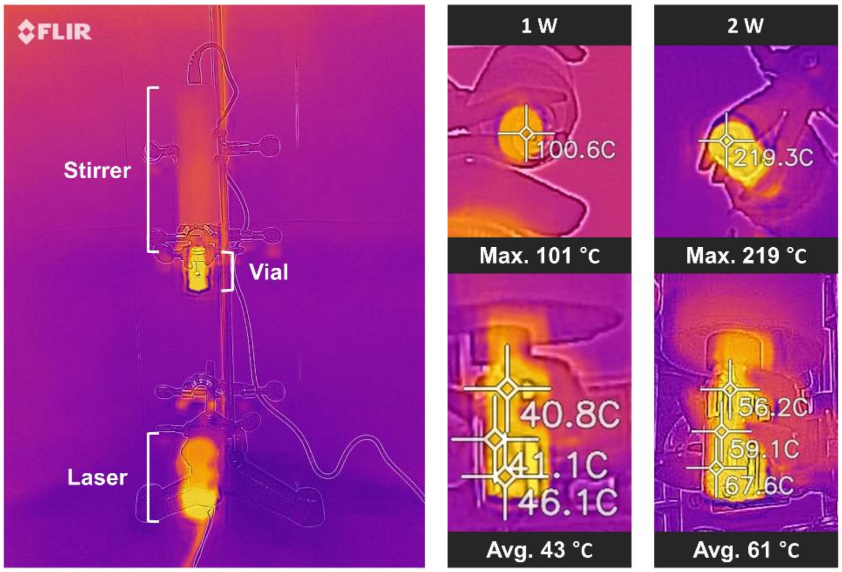

Fig. S34.

Thermographic images of (left) the overall photothermal conversion system, (right-top) the bottom of the vial containing DGIST-7@PDMS, and (right-bottom) the solution for the Knoevenagel condensation during (left) 1 W and (right) 2 W IR laser irradiation.

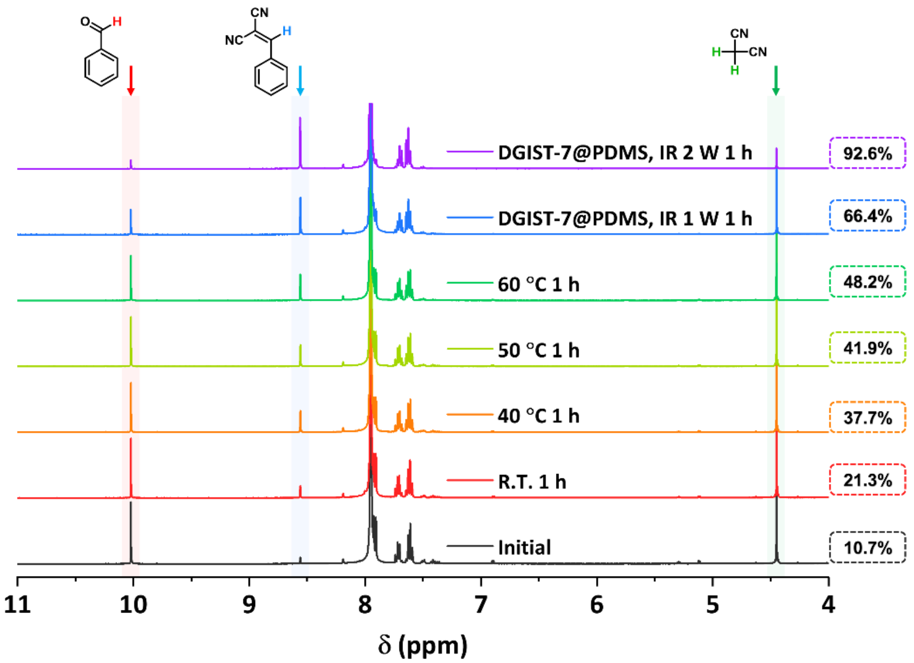

Fig. S35.

NMR spectra of the solutions after the Knoevenagel condensation under the six heating conditions. The numbers in the dotted boxes indicate the conversion yields for each condition.

Data S1. (separate file)
Crystallographic information file for DGIST-6.

Data S2. (separate file)
Crystallographic information file for DGIST-7.

Data S3. (separate file)
Crystallographic information file for DGIST-7-[Cu^{2+}].

Data S4. (separate file)
Crystallographic information file for oxi-DGIST-7.

Data S5. (separate file)
Crystallographic information file for Li^{+}@DGIST-7.

REFERENCES AND NOTES

1. M. Al Kobaisi, S. V. Bhosale, K. Latham, A. M. Raynor, S. V. Bhosale, Functional naphthalene diimides: Synthesis, properties, and applications. *Chem. Rev.* **116**, 11685–11796 (2016).

2. S. V. Bhosale, M. Al Kobaisi, R. W. Jadhav, P. P. Morajkar, L. A. Jones, S. George, Naphthalene diimides: Perspectives and promise. *Chem. Soc. Rev.* **50**, 9845–9998 (2021).

3. S. Guha, S. Saha, Fluoride ion sensing by an anion−π interaction. *J. Am. Chem. Soc.* **132**, 17674–17677 (2010).

4. Y. Takashima, V. M. Martínez, S. Furukawa, M. Kondo, S. Shimomura, H. Uehara, M. Nakahama, K. Sugimoto, S. Kitagawa, Molecular decoding using luminescence from an entangled porous framework. *Nat. Commun.* **2**, 168 (2011).

5. Y. Liang, Z. Chen, Y. Jing, Y. Rong, A. Facchetti, Y. Yao, Heavily n-dopable π-conjugated redox polymers with ultrafast energy storage capability. *J. Am. Chem. Soc.* **137**, 4956–4959 (2015).

6. H. Abe, A. Kawasaki, T. Takeda, N. Hoshino, W. Matsuda, S. Seki, T. Akutagawa, Crystal lattice design of H_2O-tolerant n-type semiconducting dianionic naphthalenediimide derivatives. *J. Am. Chem. Soc.* **143**, 1046–1060 (2021).

7. A. M. Evans, K. A. Collins, S. Xun, T. G. Allen, S. Jhulki, I. Castano, H. L. Smith, M. J. Strauss, A. K. Oanta, L. Liu, L. Sun, O. G. Reid, G. Sini, D. Puggioni, J. M. Rondinelli, T. Rajh, N. C. Gianneschi, A. Kahn, D. E. Freedman, H. Li, S. Barlow, G. Rumbles, J.-L. Brédas, S. R. Marder, W. R. Dichtel, Controlled n-doping of naphthalene-diimide-based 2D polymers. *Adv. Mater.* **34**, 2101932 (2021).

8. E. Castaldelli, K. D. G. Imalka Jayawardena, D. C. Cox, G. J. Clarkson, R. I. Walton, L. Le-Quang, J. Chauvin, S. R. P. Silva, G. J.-F. Demets, Electrical semiconduction modulated by light in a cobalt and naphthalene diimide metal-organic framework. *Nat. Commun.* **8**, 2139 (2017).

9. Y. Jiao, K. Liu, G. Wang, Y. Wang, X. Zhang, Supramolecular free radicals: Near-infrared organic materials with enhanced photothermal conversion. *Chem. Sci.* **6**, 3975–3980 (2015).

10. Y. Lv, F. Li, S. Wang, G. Lu, W. Bao, Y. Wang, Z. Tian, W. Wei, G. Ma, Near-infrared light–triggered platelet arsenal for combined photothermal-immunotherapy against cancer. *Sci. Adv.* **7**, eabd7614 (2021).

11. Y. Zhao, C. Beuchat, Y. Domoto, J. Gajewy, A. Wilson, J. Mareda, N. Sakai, S. Matile, Anion−π catalysis. *J. Am. Chem. Soc.* **136**, 2101–2111 (2014).

12. S. Kumar, M. R. Ajayakumar, G. Hundal, P. Mukhopadhyay, Extraordinary stability of naphthalenediimide radical ion and its ultra-electron-deficient precursor: Strategic role of the phosphonium group. *J. Am. Chem. Soc.* **136**, 12004–12010 (2014).

13. T. Jiao, K. Cai, J. N. Nelson, Y. Jiao, Y. Qiu, G. Wu, J. Zhou, C. Cheng, D. Shen, Y. Feng, Z. Liu, M. R. Wasielewski, J. F. Stoddart, H. Li, Stabilizing the naphthalenediimide radical within a tetracationic cyclophane. *J. Am. Chem. Soc.* **141**, 16915–16922 (2019).

14. A.-B. Bornhof, A. Bauzá, A. Aster, M. Pupier, A. Frontera, E. Vauthey, N. Sakai, S. Matile, Synergistic anion–$(\pi)_n$–π catalysis on π-stacked foldamers. *J. Am. Chem. Soc.* **140**, 4884–4892 (2018).

15. S. K. Keshri, T. Ishizuka, T. Kojima, Y. Matsushita, M. Takeuchi, Long-range order in supramolecular π assemblies in discrete multidecker naphthalenediimides. *J. Am. Chem. Soc.* **143**, 3238–3244 (2021).

16. W. S. Jeon, H.-J. Kim, C. Lee, K. Kim, Control of the stoichiometry in host–guest complexation by redox chemistry of guests: Inclusion of methylviologen in cucurbit[8]uril. *Chem. Commun.*, **17** 1828–1829 (2002).

17. A. Y. Ziganshina, Y. H. Ko, W. S. Jeon, K. Kim, Stable π-dimer of a tetrathiafulvalene cation radical encapsulated in the cavity of cucurbit[8]uril. *Chem. Commun.*, **7** 806–807 (2004).

18. X. Zhao, F. Liu, Z. Zhao, H. Karoui, D. Bardelang, O. Ouari, S. Liu, Effects of cucurbit[*n*]uril (*n* = 7, 8, 10) hosts on the formation and stabilization of a naphthalenediimide (NDI) radical anion. *Org. Biomol. Chem.* **16**, 3809–3815 (2018).

19. A. C. Fahrenbach, J. C. Barnes, D. A. Lanfranchi, H. Li, A. Coskun, J. J. Gassensmith, Z. Liu, D. Benítez, A. Trabolsi, W. A. Goddard, M. Elhabiri, J. F. Stoddart, Solution-phase mechanistic study and solid-state structure of a tris(bipyridinium radical cation) inclusion complex. *J. Am. Chem. Soc.* **134**, 3061–3072 (2012).

20. Y. Wu, M. Frasconi, D. M. Gardner, P. R. McGonigal, S. T. Schneebeli, M. R. Wasielewski, J. F. Stoddart, Electron delocalization in a rigid cofacial naphthalene-1,8:4,5-bis(dicarboximide) dimer. *Angew. Chem. Int. Ed.* **53**, 9476–9481 (2014).

21. S. K. Keshri, A. Takai, T. Ishizuka, T. Kojima, M. Takeuchi, Conformational dynamics of monomer- versus dimer-like features in a naphthalenediimide-based conjugated cyclophane. *Angew. Chem. Int. Ed.* **59**, 5254–5258 (2020).

22. H.-C. Zhou, J. R. Long, O. M. Yaghi, Introduction to metal–organic frameworks. *Chem. Rev.* **112**, 673–674 (2012).

23. H. Furukawa, K. E. Cordova, M. O'Keeffe, O. M. Yaghi, The chemistry and applications of metal-organic frameworks. *Science* **341**, 1230444 (2013).

24. Q. Chen, J. Sun, P. Li, I. Hod, P. Z. Moghadam, Z. S. Kean, R. Q. Snurr, J. T. Hupp, O. K. Farha, J. F. Stoddart, A redox-active bistable molecular switch mounted inside a metal–organic framework. *J. Am. Chem. Soc.* **138**, 14242–14245 (2016).

25. X. Chen, H. Xie, E. R. Lorenzo, C. J. Zeman, Y. Qi, Z. H. Syed, A. E. B. S. Stone, Y. Wang, S. Goswami, P. Li, T. Islamoglu, E. A. Weiss, J. T. Hupp, G. C. Schatz, M. R. Wasielewski, O. K. Farha, Direct observation of modulated radical spin states in metal–organic frameworks by controlled flexibility. *J. Am. Chem. Soc.* **144**, 2685–2693 (2022).

26. H. He, L. Hashemi, M.-L. Hu, A. Morsali, The role of the counter-ion in metal-organic frameworks' chemistry and applications. *Coord. Chem. Rev.* **376**, 319–347 (2018).

27. H. Yuan, N. Li, W. Fan, H. Cai, D. Zhao, Metal-organic framework based gas sensors. *Adv. Sci.* **9**, 2104374 (2022).

28. A. Mallick, B. Garai, M. A. Addicoat, P. S. Petkov, T. Heine, R. Banerjee, Solid state organic amine detection in a photochromic porous metal organic framework. *Chem. Sci.* **6**, 1420–1425 (2015).

29. B. Garai, A. Mallick, R. Banerjee, Photochromic metal–organic frameworks for inkless and erasable printing. *Chem. Sci.* **7**, 2195–2200 (2016).

30. Y.-X. Tan, S.-X. Lin, C. Liu, Y. Huang, M. Zhou, Q. Kang, D. Yuan, M. Hong, Boosting photocatalytic cross-dehydrogenative coupling reaction by incorporating $[Ru^{II}(bpy)_3]$ into a radical metal-organic framework. *Appl. Catal.* **227**, 425–432 (2018).

31. J.-Z. Liao, H.-L. Zhang, S.-S. Wang, J.-P. Yong, X.-Y. Wu, R. Yu, C.-Z. Lu, Multifunctional radical-doped polyoxometalate-based host–guest material: Photochromism and photocatalytic activity. *Inorg. Chem.* **54**, 4345–4350 (2015).

32. J.-Z. Liao, C. Wu, X.-Y. Wu, S.-Q. Deng, C.-Z. Lu, Exceptional photosensitivity of a polyoxometalate-based charge-transfer hybrid material. *Chem. Commun.* **52**, 7394–7397 (2016).

33. B. Ding, B. Chan, N. Proschogo, M. B. Solomon, C. J. Kepert, D. M. D'Alessandro, A cofacial metal–organic framework-based photocathode for carbon dioxide reduction. *Chem. Sci.* **12**, 3608–3614 (2021).

34. J.-J. Liu, J.-J. Fu, T. Liu, X. Shen, F.-X. Cheng, The modulation effect of an electron-rich guest on the luminescence of naphthalene diimide-based metal–organic frameworks. *Inorg. Chem. Front.* **9**, 3898–3906 (2022).

35. C. F. Leong, B. Chan, T. B. Faust, D. M. D'Alessandro, Controlling charge separation in a novel donor–acceptor metal–organic framework *via* redox modulation. *Chem. Sci.* **5**, 4724–4728 (2014).

36. N. Sikdar, K. Jayaramulu, V. Kiran, K. V. Rao, S. Sampath, S. J. George, T. K. Maji, Redox-active metal–organic frameworks: Highly stable charge-separated states through strut/guest-to-strut electron transfer. *Chem. A Eur. J.* **21**, 11701–11706 (2015).

37. J. He, Q. Han, J. Li, Z. Shi, X. Shi, J. Niu, Ternary supramolecular system for photocatalytic oxidation with air by consecutive photo-induced electron transfer processes. *J. Catal.* **376**, 161–167

(2019).

38. J. He, J. Li, Q. Han, C. Si, G. Niu, M. Li, J. Wang, J. Niu, Photoactive metal–organic framework for the reduction of aryl halides by the synergistic effect of consecutive photoinduced electron-transfer and hydrogen-atom-transfer processes. *ACS Appl. Mater. Interfaces* **12**, 2199–2206 (2020).

39. Z. Guo, D. K. Panda, M. A. Gordillo, A. Khatun, H. Wu, W. Zhou, S. Saha, Lowering band gap of an electroactive metal–organic framework via complementary guest intercalation. *ACS Appl. Mater. Interfaces* **9**, 32413–32417 (2017).

40. S. Goswami, J. N. Nelson, T. Islamoglu, Y.-L. Wu, O. K. Farha, M. R. Wasielewski, Photoexcited naphthalene diimide radical anion linking the nodes of a metal–organic framework: A heterogeneous super-reductant. *Chem. Mater.* **30**, 2488–2492 (2018).

41. C. Hua, P. W. Doheny, B. Ding, B. Chan, M. Yu, C. J. Kepert, D. M. D'Alessandro, Through-space intervalence charge transfer as a mechanism for charge delocalization in metal–organic frameworks. *J. Am. Chem. Soc.* **140**, 6622–6630 (2018).

42. B. Ding, C. Hua, C. J. Kepert, D. M. D'Alessandro, Influence of structure–activity relationships on through-space intervalence charge transfer in metal–organic frameworks with cofacial redox-active units. *Chem. Sci.* **10**, 1392–1400 (2019).

43. P. W. Doheny, J. K. Clegg, F. Tuna, D. Collison, C. J. Kepert, D. M. D'Alessandro, Quantification of the mixed-valence and intervalence charge transfer properties of a cofacial metal–organic framework *via* single crystal electronic absorption spectroscopy. *Chem. Sci.* **11**, 5213–5220 (2020).

44. K.-Q. Hu, P.-X. Qiu, L.-W. Zeng, S.-X. Hu, L. Mei, S.-W. An, Z.-W. Huang, X.-H. Kong, J.-H. Lan, J.-P. Yu, Z.-H. Zhang, Z.-F. Xu, J. K. Gibson, Z.-F. Chai, Y.-F. Bu, W.-Q. Shi, Solar-driven nitrogen fixation catalyzed by stable radical-containing mOFs: Improved efficiency induced by a structural transformation. *Angew. Chem. Int. Ed.* **59**, 20666–20671 (2020).

45. S. S. Park, C. H. Hendon, A. J. Fielding, A. Walsh, M. O'Keeffe, M. Dincă, The organic secondary building unit: Strong intermolecular π interactions define topology in MIT-25, a mesoporous MOF with proton-replete channels. *J. Am. Chem. Soc.* **139**, 3619–3622 (2017).

46. M. J. Kalmutzki, N. Hanikel, O. M. Yaghi, Secondary building units as the turning point in the development of the reticular chemistry of MOFs. *Sci. Adv.* **4**, eaat9180 (2018).

47. J.-H. Deng, J. Luo, Y.-L. Mao, S. Lai, Y.-N. Gong, D.-C. Zhong, T.-B. π-π stacking interactions: Non-negligible forces for stabilizing porous supramolecular frameworks. *Sci. Adv.* **6**, eaax9976 (2020).

48. X.-L. Lv, L. Feng, K.-Y. Wang, L.-H. Xie, T. He, W. Wu, J.-R. Li, H.-C. Zhou, A series of mesoporous rare-earth metal–organic frameworks constructed from organic secondary building units. *Angew. Chem. Int. Ed.* **60**, 2053–2057 (2021).

49. Y. Tan, Z. Fu, Y. Zeng, H. Chen, S. Liao, J. Zhang, J. Dai, Highly stable photochromic crystalline material based on a close-packed layered metal–viologen coordination polymer. *J. Mater. Chem.* **22**, 17452–17455 (2012).

50. S.-L. Li, M. Han, Y. Zhang, G.-P. Li, M. Li, G. He, X.-M. Zhang, X-ray and UV dual photochromism, thermochromism, electrochromism, and amine-selective chemochromism in an anderson-like Zn_7 Cluster-Based 7-fold interpenetrated framework. *J. Am. Chem. Soc.* **141**, 12663–12672 (2019).

51. S. Guha, F. S. Goodson, S. Roy, L. J. Corson, C. A. Gravenmier, S. Saha, Electronically regulated thermally and light-gated electron transfer from anions to naphthalenediimides. *J. Am. Chem. Soc.* **133**, 15256–15259 (2011).

52. S. Guha, F. S. Goodson, L. J. Corson, S. Saha, Boundaries of anion/naphthalenediimide interactions: From anion−π interactions to anion-induced charge-transfer and electron-transfer phenomena. *J. Am. Chem. Soc.* **134**, 13679–13691 (2012).

53. S. Saha, Anion-induced electron transfer. *Acc. Chem. Res.* **51**, 2225–2236 (2018).

54. J. Su, S. Yuan, H.-Y. Wang, L. Huang, J.-Y. Ge, E. Joseph, J. Qin, T. Cagin, J.-L. Zuo, H.-C. Zhou, Redox-switchable breathing behavior in tetrathiafulvalene-based metal–organic frameworks. *Nat. Commun.* **8**, 2008 (2017).

55. L. Qu, H. Iguchi, S. Takaishi, F. Habib, C. F. Leong, D. M. D'Alessandro, T. Yoshida, H. Abe, E. Nishibori, M. Yamashita, Porous molecular conductor: Electrochemical fabrication of through-space conduction pathways among linear coordination polymers. *J. Am. Chem. Soc.* **141**, 6802–6806 (2019).

56. A. K. Cheetham, G. Kieslich, H. H.-M. Yeung, Thermodynamic and kinetic effects in the crystallization of metal–organic frameworks. *Acc. Chem. Res.* **51**, 659–667 (2018).

57. X. Gong, H. Noh, N. C. Gianneschi, O. K. Farha, Interrogating kinetic versus thermodynamic topologies of metal–organic frameworks via combined transmission electron microscopy and X-ray diffraction analysis. *J. Am. Chem. Soc.* **141**, 6146–6151 (2019).

58. C. A. Hunter, J. K. M. Sanders, The nature of .pi.-.pi. interactions. *J. Am. Chem. Soc.* **112**, 5525–5534 (1990).

59. A. Takai, T. Yasuda, T. Ishizuka, T. Kojima, M. Takeuchi, A directly linked ferrocene–naphthalenediimide conjugate: Precise control of stacking structures of π-systems by redox stimuli. *Angew. Chem. Int. Ed.* **52**, 9167–9171 (2013).

60. Y. Ye, Z. Ma, L. Chen, H. Lin, Q. Lin, L. Liu, Z. Li, S. Chen, Z. Zhang, S. Xiang, Microporous metal–organic frameworks with open metal sites and π-Lewis acidic pore surfaces for recovering ethylene from polyethylene off-gas. *J. Mater. Chem. A* **6**, 20822–20828 (2018).

61. S. Park, J. Lee, H. Jeong, S. Bae, J. Kang, D. Moon, J. Park, Multi-stimuli-engendered radical-anionic MOFs: Visualization of structural transformation upon radical formation. *Chem.* **8**, 1993–2010 (2022).

62. Y. Kumar, S. Kumar, K. Mandal, P. Mukhopadhyay, Isolation of tetracyano-naphthalenediimide and its stable planar radical anion. *Angew. Chem. Int. Ed.* **57**, 16318–16322 (2018).

63. E. A. Dolgopolova, O. A. Ejegbavwo, C. R. Martin, M. D. Smith, W. Setyawan, S. G. Karakalos, C. H. Henager, H.-C. zur Loye, N. B. Shustova, Multifaceted modularity: A key for stepwise building of hierarchical complexity in actinide metal–organic frameworks. *J. Am. Chem. Soc.* **139**, 16852–16861 (2017).

64. D. Sheng, L. Zhu, C. Xu, C. Xiao, Y. Wang, Y. Wang, L. Chen, J. Diwu, J. Chen, Z. Chai, T. E. Albrecht-Schmitt, S. Wang, Efficient and selective uptake of TcO_4^- by a cationic metal–organic framework material with open Ag^+ sites. *Environ. Sci. Technol.* **51**, 3471–3479 (2017).

65. K.-i. Otake, Y. Cui, C. T. Buru, Z. Li, J. T. Hupp, O. K. Farha, Single-atom-based vanadium oxide catalysts supported on metal–organic frameworks: Selective alcohol oxidation and structure–activity relationship. *J. Am. Chem. Soc.* **140**, 8652–8656 (2018).

66. K.-i. Otake, J. Ye, M. Mandal, T. Islamoglu, C. T. Buru, J. T. Hupp, M. Delferro, D. G. Truhlar, C. J. Cramer, O. K. Farha, Enhanced activity of heterogeneous Pd(II) catalysts on acid-functionalized metal–organic frameworks. *ACS Catal.* **9**, 5383–5390 (2019).

67. X.-N. Wang, P. Zhang, A. Kirchon, J.-L. Li, W.-M. Chen, Y.-M. Zhao, B. Li, H.-C. Zhou, Crystallographic visualization of postsynthetic nickel clusters into metal–organic framework. *J. Am. Chem. Soc.* **141**, 13654–13663 (2019).

68. Y.-X. Xie, W.-N. Zhao, G.-C. Li, P.-F. Liu, L. Han, A naphthalenediimide-based metal–organic framework and thin film exhibiting photochromic and electrochromic properties. *Inorg. Chem.* **55**, 549–551 (2016).

69. Y. Wang, W. Zhu, W. Du, X. Liu, X. Zhang, H. Dong, W. Hu, Cocrystals strategy towards materials for near-infrared photothermal conversion and imaging. *Angew. Chem. Int. Ed.* **57**, 3963–3967 (2018).

70. J.-D. Xiao, H.-L. Jiang, Metal–organic frameworks for photocatalysis and photothermal catalysis. *Acc. Chem. Res.* **52**, 356–366 (2019).

71. Y. Lu, D. Liu, Y.-J. Lin, G.-X. Jin, A hierarchical assembly strategy for near-infrared photothermal conversion: Unconventional heterogeneous metalla[2]catenanes. *Chem. Sci.* **11**, 11509–11513 (2020).

72. B. Lü, Y. Chen, P. Li, B. Wang, K. Müllen, M. Yin, Stable radical anions generated from a porous perylenediimide metal-organic framework for boosting near-infrared photothermal conversion. *Nat. Commun.* **10**, 767 (2019).

73. K. van Beurden, S. de Koning, D. Molendijk, J. van Schijndel, The Knoevenagel reaction: A review of the unfinished treasure map to forming carbon–carbon bonds. *Green Chem. Lett. Rev.* **13**, 349–364 (2020).

74. J. P. Perdew, K. Burke, M. Ernzerhof, Generalized gradient approximation made simple. *Phys. Rev. Lett.* **77**, 3865–3868 (1996).

75. P. E. Blöchl, Projector augmented-wave method. *Phys. Rev.* **50**, 17953–17979 (1994).

76. G. Kresse, J. Furthmüller, Efficient iterative schemes for *ab initio* total-energy calculations using a plane-wave basis set. *Phys. Rev.* **54**, 11169–11186 (1996).

77. G. Kresse, D. Joubert, From ultrasoft pseudopotentials to the projector augmented-wave method. *Phys. Rev.* **59**, 1758–1775 (1999).

78. S. Grimme, Semiempirical GGA-type density functional constructed with a long-range dispersion correction. *J. Comput. Chem.* **27**, 1787–1799 (2006).

79. L. Han, L. Qin, L. Xu, Y. Zhou, J. Sun, X. Zou, A novel photochromic calcium-based metal–organic framework derived from a naphthalene diimide chromophore. *Chem. Commun.* **49**, 406–408 (2013).

80. J.-Z. Liao, J.-F. Chang, L. Meng, H.-L. Zhang, S.-S. Wang, C.-Z. Lu, Lone pair-π interaction-induced generation of photochromic coordination networks with photoswitchable conductance. *Chem. Commun.* **53**, 9701–9704 (2017).

81. X. Shang, I. Song, G. Y. Jung, W. Choi, H. Ohtsu, J. H. Lee, J. Y. Koo, B. Liu, J. Ahn, M. Kawano, S. K. Kwak, J. H. Oh, Chiral self-sorted multifunctional supramolecular biocoordination polymers and their applications in sensors. *Nat. Commun.* **9**, 3933 (2018).

82. X. Shang, I. Song, G. Y. Jung, W. Choi, H. Ohtsu, J. H. Lee, J. Ahn, J. Y. Koo, M. Kawano, S. K. Kwak, J. H. Oh, Micro−/nano-sized multifunctional heterochiral metal–organic frameworks for high-performance visible–blind UV photodetectors. *J. Mater. Chem. C* **9**, 7310–7318 (2021).

83. B. A. Johnson, A. Bhunia, H. Fei, S. M. Cohen, S. Ott, Development of a UiO-type thin film

electrocatalysis platform with redox-active linkers. *J. Am. Chem. Soc.* **140**, 2985–2994 (2018).

84. F. J. Rizzuto, T. B. Faust, B. Chan, C. Hua, D. M. D'Alessandro, C. J. Kepert, Experimental and computational studies of a multi-electron donor–acceptor ligand containing the thiazolo[5,4-*d*]thiazole core and its incorporation into a metal–organic framework. *Chem. A Eur. J.* **20**, 17597–17605 (2014).

85. J. Ren, Y. Meng, X. Zhang, Y. Gao, L. Liu, X. Zhou, Z. Zhang, L. Zeng, J. Ke, Self-assembled perylene diimide modified NH_2-UiO-66 (Zr) construct n-n heterojunction catalysts for enhanced Cr (VI) photocatalytic reduction. *Sep. Purif. Technol.* **296**, 121423 (2022).

86. J. W. Shin, K. Eom, D. Moon, BL2D-SMC, the supramolecular crystallography beamline at the Pohang Light Source II, Korea, *J. Synchrotron. Radiat.* **23**, 369–373 (2016).

87. Z. Otwinowski, W. Minor,"Processing of x-ray diffraction data collected in oscillation mode" in *Methods* Enzymol (Academic Press, 1997), vol. 276, pp. 307–326.

88. G. M. Sheldrick, Crystal structure refinement with SHELXL. *Acta Crystallogr. C Struct. Chem.* **71**, 3–8 (2015).
